# Environment-Driven Variability in Absolute Band Edge Positions and Work Functions of Reduced Ceria

**DOI:** 10.1021/jacs.4c05053

**Published:** 2024-06-05

**Authors:** Xingfan Zhang, Christopher Blackman, Robert G. Palgrave, Sobia Ashraf, Avishek Dey, Matthew O. Blunt, Xu Zhang, Taifeng Liu, Shijia Sun, Lei Zhu, Jingcheng Guan, You Lu, Thomas W. Keal, John Buckeridge, C. Richard A. Catlow, Alexey A. Sokol

**Affiliations:** † Kathleen Lonsdale Materials Chemistry, Department of Chemistry, University College London, London WC1H 0AJ, U.K.; ‡ Department of Chemistry, University College London, Christopher Ingold Building, 20 Gordon Street, London WC1H 0AJ, U.K.; § Scientific Computing Department, STFC Daresbury Laboratory, Warrington WA4 4AD, Cheshire, U.K.; ∥ School of Engineering, London South Bank University, London SE1 OAA, U.K.; ⊥ School of Chemistry, Cardiff University, Park Place, Cardiff CF10 1AT, U.K.; # School of Chemical Engineering and Technology, Tianjin University, Tianjin 300350, P. R. China; ∇ National & Local Joint Engineering Research Center for Applied Technology of Hybrid Nanomaterials, Henan University, Kaifeng 475004, China

## Abstract

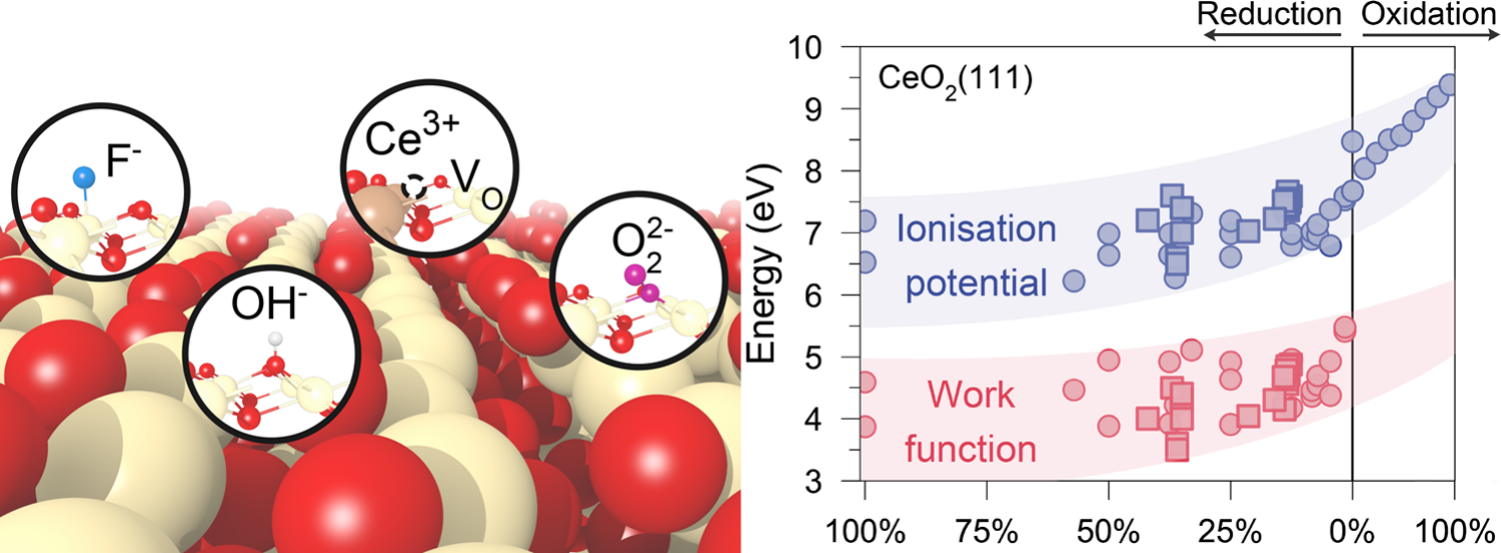

The absolute band edge positions and work function (Φ) are the key electronic properties of metal oxides that determine their performance in electronic devices and photocatalysis. However, experimental measurements of these properties often show notable variations, and the mechanisms underlying these discrepancies remain inadequately understood. In this work, we focus on ceria (CeO_2_), a material renowned for its outstanding oxygen storage capacity, and combine theoretical and experimental techniques to demonstrate environmental modifications of its ionization potential (IP) and Φ. Under O-deficient conditions, reduced ceria exhibits a decreased IP and Φ with significant sensitivity to defect distributions. In contrast, the IP and Φ are elevated in O-rich conditions due to the formation of surface peroxide species. Surface adsorbates and impurities can further augment these variabilities under realistic conditions. We rationalize the shifts in energy levels by separating the individual contributions from bulk and surface factors, using hybrid quantum mechanical/molecular mechanical (QM/MM) embedded-cluster and periodic density functional theory (DFT) calculations supported by interatomic-potential-based electrostatic analyses. Our results highlight the critical role of on-site electrostatic potentials in determining the absolute energy levels in metal oxides, implying a dynamic evolution of band edges under catalytic conditions.

## 1 Introduction

Ceria (CeO_2_) is a rare-earth oxide widely used in heterogeneous catalysis due to its exceptional oxygen storage and catalyst-stabilizing capacities. The successful application of ceria in automotive three-way catalysts has attracted extensive research into broader applications, such as reforming processes, CO_2_ conversion, and photocatalysis.^1,2^ The catalytic versatility of ceria is largely attributable to its dynamical oxygen cycling, facilitated by mobile oxygen vacancies (V_O_) and a reversible Ce^4+^/Ce^3+^ oxidation state,^3^ endowing ceria-based catalysts with nonstoichiometric flexibility and complex surface chemistry to satisfy many applications.

Recent work has identified ceria-based catalysts as potential photocatalysts for applications ranging from organic pollutant degradation to water splitting and CO_2_ conversion.^4−6^ The photocatalytic activity of semiconductors is critically dependent on their band structure: an appropriate band gap for efficient light absorption and optimal band edge positions to align with the reaction potentials.^7^ In particular, the absolute band edge positions of photocatalysts govern whether the target chemical reactions can occur. For example, to initiate photocatalytic water splitting, the conduction band minimum (CBM) and valence band maximum (VBM) of the photocatalyst must align with the redox potential of water.^8^ Hence, knowledge of the absolute band edge positions of a photocatalyst with respect to the vacuum level in operating conditions is of great importance to optimize their optical and electrical properties. Moreover, the absolute band edge positions of metal oxides determine the alignment of energy levels, affecting charge carrier injection, transport, and extraction, which are crucial for the development of electronic and optoelectronic devices, including light-emitting diodes, photovoltaics, and transistors.^9,10^ Accurate knowledge of these properties enables the optimization of material interfaces when integrated into the multilayered device architecture, which significantly affects the overall efficiency and stability.^11,12^

Adjusting the band gap of ceria-based photocatalysts is feasible through modifications in stoichiometry,^13^ morphology,^14^ nanoparticle size,^15^ and doping,^16^ offering avenues to enhance the visible light reactivity. The integration of ceria into heterojunctions has also shown improved photocatalytic efficiency by facilitating electron–hole separation.^5^ To maximize photocatalytic performance, a thorough understanding of the fundamental band alignment among different materials is necessary, which affects the distribution and mobility of charge carriers and thereby determines the overall performance.

In the solid state, the absolute band edge positions and work function (Φ) are sensitive to surface conditions and can vary significantly among individual samples or under different environments. Klein et al.^17,18^ observed variations of ca. 1 eV across the ionization potentials (IPs) of transparent conducting oxide (ZnO, In_2_O_3_, and SnO_2_) thin films due to differences in their crystal orientations, morphologies, and defect concentrations. Zilberberg et al.^19^ observed an IP reduction of 1.6 eV for V_2_O_5_ prepared in an ultrahigh vacuum after exposure to air. CeO_2_ stands out as an exceptional case due to the substantial differences in surface potential measurements, ranging from 5.47 to 9.1 eV for IP and from 3.5 to 6.3 eV for Φ.^15,20−24^ To understand the origin of these experimental variations, we have recently provided theoretical insights into the bulk and surface contributions to IPs of CeO_2_ and other metal oxides.^25^ By combining electrostatic analyses with electronic-structure calculations, we have elucidated the roles of orientation-dependent stacking sequence, surface polarization, and structural relaxation in modifying the local electrostatic environments, thereby inducing shifts in the absolute band edge positions. These findings offer a clear explanation for the experimentally observed orientation dependence of IPs in metal oxides.

Our previous work concentrated on the intrinsic surface orientation effects in stoichiometric oxides without accounting for the variable surface chemistry and stoichiometry, which could also play a vital role in determining the electronic properties of easily reducible oxides like ceria. The formation energies of oxygen vacancies near the surfaces of CeO_2_ are considerably lower than those in bulk,^26,27^ leading to various types of surface reconstruction in substoichiometric phases.^28,29^ Ceria shows nonstoichiometric behavior at elevated temperatures, while a continuous series of oxygen-deficient ordered CeO_2–*x*_ (*x* = 0–0.5) phases have been detected experimentally, including Ce_2_O_3_, Ce_7_O_12_, and Ce_11_O_20_.^30−32^^30−32^ However, an excess of oxygen in the environment can refill the near-surface vacancies, and even form peroxide species, particularly in oxygen-plasma-treated samples.^33,34^ The variation of the absolute band edge positions in CeO_2–*x*_ with environmental changes, though crucial to the photocatalytic performance, remains inadequately explored. From previous studies, the enhanced photocatalytic performance of ceria by surface oxygen vacancies has been reported and attributed mainly to the narrowed band gap, improved charge separation, and promoted catalytic redox reactions,^35,36^ with little knowledge of effects on the absolute band edge positions.

In this work, we combined three computational techniques, namely, plane-wave density functional theory (DFT) calculations, hybrid quantum mechanical/molecular mechanical (QM/MM) embedded-cluster calculations, and interatomic-potential-based electrostatic analyses. These theoretical studies are further complemented by experimental characterizations using X-ray photoelectron spectroscopy (XPS) and ultraviolet photoelectron spectroscopy (UPS) on thin-film samples of reduced ceria, providing a clear understanding of the variations in band structures of easily reducible oxides in different environments and their underlying mechanisms.

## 2 Methods

### 2.1 Plane-Wave Density Functional Theory (DFT) Calculations

Spin-polarized DFT calculations were performed using the Vienna Ab-initio Simulation Package (VASP) code.^37^ A plane-wave basis set with a cutoff energy of 600 eV was employed. The projector augmented wave (PAW)^38^ method was used to describe interactions between the core and valence electrons, considering Ce (5s, 5p, 4f, 5d, 6s) and O (2s, 2p) as valence states. Monkhorst–Pack *k*-point meshes with a density of 0.04 × 2π Å^–1^ were generated for bulk systems. The energy convergence criterion in electronic steps was set as 10^–6^ eV, and structural optimizations adopted a force criterion of 10^–2^ eV Å^–1^ on each ion.

DFT calculations were performed at several levels of theory. First, the Perdew–Burke–Ernzerhof (PBE) exchange–correlation functional^39^ was used in conjunction with a Hubbard correction scheme^40^ (PBE + *U*, *U*_Ce 4f_ = 5 eV) for the Ce 4f orbitals to capture the localized nature of 4f electrons in CeO_2–*x*_.^26,41^ Additionally, hybrid functionals, including PBE0^42^ and HSE06^43^ with 25% nonlocal Hartree–Fock (HF) exchange, were also employed. The performance of these methods in describing the lattice structure and band gap of various CeO_2–*x*_ phases was compared and shown in Table S1. The calculated lattice parameters for CeO_2_ and its reduced phases by these hybrid functionals align closely with low-temperature experimental data, with HSE06 predictions for band gaps showing slightly improved accuracy. PBE + *U* predictions overestimate the ground-state lattice parameters by approximately 2% (5.490 Å by PBE + *U* compared with experimental measurement of 5.395 Å extrapolated to 0 K^26,44−47^) and underestimate the band gap (2.31 eV by PBE + *U* compared with 4 eV from experiment^48^) of CeO_2_. We noted that while the O_2p_ → Ce_4f_ band gap of CeO_2_ is commonly reported as 3.0–3.4 eV,^1^ Pelli Cresi et al.^48^ recently proposed a revised optical gap of 4 eV. Ultrafast transient absorption spectra assigned the 3–4 eV absorption region as the Urbach tail with implications for the ultrafast Ce^3+^ polaron formation. Hence, the 3–3.4 eV band gap obtained through steady-state measurements via the Tauc method actually represents the position of occupied Ce_4f_ polaronic states.

In periodic slab models, the surface IP is calculated by
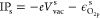
1where *V*_vac_^s^ is the average electrostatic Hartree potential at the central plane of the vacuum region and ϵ_O_2p__^s^ denotes the highest occupied O_2p_ energy level. The predicted IP of pristine CeO_2_(111) at the PBE0 level of theory is 7.67 eV, which is in excellent agreement with experimental measurements of 7.7 eV on the stoichiometric (111)-oriented film.^20^ In contrast, the PBE + *U* scheme underestimated the IP by 1 eV. The Fermi level in semiconductors and insulators depends on concentrations of charge carriers and is very sensitive to defect and surface states. In reduced CeO_2–*x*_ systems, DFT calculations show the highest occupied state on Ce^3+^ (ϵ_Ce 4f_^s^), which can be regarded as an approximation to the Fermi level. As the work function Φ is defined as the minimum required energy to remove an electron from the Fermi level to the vacuum level, it can be calculated as
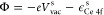
2While hybrid functionals provide a better solution to the electron self-interaction error in DFT than the DFT + *U* scheme and yield more accurate energy levels in oxide materials, they operate with computational costs that are an order of magnitude higher. To balance the accuracy and efficiency in modeling the complex atomic configurations and surface chemistry of reduced ceria, which typically comprise 100–400 atoms, we performed structural optimizations at the PBE + *U* (*U*_Ce 4f_ = 5 eV) level of theory, followed by single-point calculations using PBE0 to improve the description of the band edge positions (PBE0@PBE + *U*). Monkhorst–Pack *k*-point meshes were generated with a density of 0.06 × 2π Å^–1^ for surface models. While the overestimation of the lattice constant of CeO_2_ by PBE + *U* is expected to reduce the bulk Madelung potential at O sites by 0.37 V, the PBE0@PBE + *U* approximation results in only minor errors (less than 0.1 V) for the calculated band edge positions compared to direct PBE0 assessments, suggesting that there could be enhanced surface polarisation or relaxation that compensate for the errors associated with the lattice constant. Dipole moment correction was applied to all asymmetric slab models as implemented in the VASP code.

Our investigation mainly focused on the most stable quadrupolar (111) surface of CeO_2_. A one-sided two-region surface slab model with 24 atomic layers (8 O–Ce–O trilayers) was cleaved from the preoptimized bulk system, with the bottom half of atoms fixed during optimization to reproduce the effects of the bulk structure. A vacuum layer of 30 Å was employed in all slab models, ensuring full convergence of electrostatic potentials. Partially reduced CeO_2_(111) surfaces undergo various types of reconstruction into periodic patterns, including A-Ce_2_O_3_(1 × 1), C-Ce_2_O_3_(4 × 4), Ce_3_O_5_( × 3), Ce_3_O_5_( × ), Ce_3_O_5_(3 × 3), Ce_7_O_12_(), Ce_7_O_12_( × ), Ce_11_O_20_(), Ce_7_O_12_(), and a Ce=O terminated ( × ) reconstruction, as recently identified by Ganduglia-Pirovano et al.^28,29,49^ The magnetic interactions among Ce^3+^ ions in reduced ceria are weak, showing little energy difference between the ferromagnetic and antiferromagnetic configurations, consistent with the experimentally reported ultralow Néel temperature (6.2 K) of Ce_2_O_3_.^50,51^ Hence, for simplicity, only high-spin (ferromagnetic) states are considered in this work for slab models of partially reduced ceria. To calculate the surface IPs of CeO_2–*x*_ ordered reduced phases, surfaces maintaining the fluorite (111) orientation were cleaved from the optimized lattices, including the two phases of Ce_2_O_3_ (A-Ce_2_O_3_(001) and C-Ce_2_O_3_(111)), Ce_5_O_9_(111), Ce_6_O_11_(001), Ce_7_O_12_(111), and Ce_11_O_20_(201̅), for a direct comparison with CeO_2_(111).

To probe the effects of surface adsorbates and impurities (including O_2_^2–^, Cl^–^, F^–^, and OH^–^) on the IP of CeO_2_, a 2 ×  extension of the primitive (111) slab was used to mitigate periodic image–image interactions. The extended pristine model consisted of five trilayers in the slab, with the bottom two trilayers fixed in the structural optimization. Similarly, a 2 × 2 extension of the primitive (110) slab was used for the CeO_2_(110) surface with a thickness of eight atomic layers, whereas the bottom four layers are fixed.

### 2.2 Electrostatic Analyses Based on Shell-model Interatomic Potentials

We performed electrostatic analyses using on-site Madelung potentials (*V*_Mad_) calculated by the method of shell-model interatomic potentials, as implemented in the General Utility Lattice Program (GULP)^52,53^ code. In the shell model, an ion is described separately by a core connected by a harmonic spring to an associated massless shell.^54,55^ Calculations of lattice energy account for the electrostatic Coulomb interaction through the Ewald^56^ and Parry^57^ summation techniques, applied to three-dimensional and two-dimensional periodic models, respectively. Short-range repulsion and dispersion interactions are determined using fitted interatomic potentials. The interatomic potential was developed previously for modeling intrinsic defects in CeO_2_ using the Mott–Littleton approach, validated against hybrid QM/MM results in accurately predicting the defect structures and energies.^26^ For this study, some parameters were modified to improve the description of reduced ceria phases, which are given in Table S2. The lattice parameters of CeO_2–*x*_ phases predicted by this set of interatomic potentials are in good agreement with available experimental measurements and DFT calculations, as shown in Table S1. This interatomic potential also shows good performance in reproducing the elastic, dielectric, and vibrational properties of A-type Ce_2_O_3_ from experimental measurements and DFT calculations, as shown in Table S4.

To elucidate the relationship between the local electrostatic environment and the absolute energy level in reduced ceria, we calculated *V*_Mad_ based on DFT-optimized bulk and surface models, allowing only electronic (modeled by the shell) relaxation. The Madelung potential for an ion A is derived from a periodic summation of the electrostatic contributions from other charged species
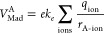
3where *k*_*e*_ is the dimensional Coulomb constant, *q*_ion_ is the charge of a surrounding ion, and *r*_A-ion_ is the distance between the surrounding ion and ion A. The *V*_Mad_ calculated in periodic bulk models is an intrinsic property of a material and allows for a direct cross-material comparison. For surface slab models, the calculated on-site Madelung potentials from different slab models were referenced relative to their corresponding vacuum-level electrostatic potential *V*_vac_, i.e.,
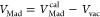
4We employed the lowest electrostatic potential values on O^2–^ (*V*_Mad_^O^) and Ce^3+^ (*V*_Mad_^Ce^3+^^) sites calculated in each model for the electrostatic analyses, which represent the upper band edges.

### 2.3 Hybrid QM/MM Embedded-Cluster Approach

A primary challenge in the theoretical evaluation of band alignment among different materials is to identify a common reference level. The hybrid QM/MM embedded-cluster approach has emerged as the appropriate methodology for determining the pure bulk contributions to the IPs of ionic materials with respect to a common reference for comparison.^25,58,59^ Within the solid-state embedding framework, the central region is treated quantum mechanically: here, we employed DFT calculations using the hybrid meta-generalized gradient approximation (meta-GGA) functional BB1K with a 42% HF exchange,^60^ consistent with our previous work.^25^ The QM region is surrounded by a cationic layer described by fitted pseudopotentials, serving as the interface bridging the MM atoms. We fitted a pseudopotential for Ce^3+^ cations based on the C-Ce_2_O_3_ embedded-cluster model (with detailed parameters given in Table S3), integrated with the previously derived Ce^4+^ pseudopotential, for interface treatment in the QM/MM models of reduced ceria.^26^ The fitting procedures systematically considered the energy gradients in each region and the spread of the deep core levels to reduce the mismatch between the QM and MM levels of theory, as implemented in the FIT_MY_ECP code (https://www.github.com/logsdail/fit_my_ecp). The rest of the model is described at the MM level of theory, using the identical shell-model potential in electrostatic analyses. The MM regions reproduce the periodic electrostatic environment inherent to ionic solids and account for the long-range polarization due to the variation of electronic states (e.g., ionization). Our QM/MM calculations were performed using the Py-ChemShell code,^61−63^ integrating QM calculations performed by NWChem^64^ and MM calculations by GULP.^52,53^

To calculate the bulk contribution to the IPs of reduced ceria, we constructed QM/MM models of various CeO_2–*x*_ phases based on the PBE0-optimized lattice structures from plane-wave DFT; QM single-point calculations accompanied by MM shell relaxations were performed to obtain the energies of the charge-neutral and ionized states. For reduced CeO_2–*x*_ phases, the energy of ionization process from the highest occupied O_2p_ state was considered as the bulk IP, which is determined by the energy difference between the ionized and charge-neutral states
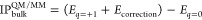
5The long-range polarization effect in charged systems is treated with an *a posteriori* correction on the QM/MM energy, as proposed by Jost^65,66^
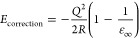
6where *R* is the radius of the active region (15 Å), *Q* is the net charge of the ionized system (+1e), and ε_∞_ is the high-frequency dielectric constant of the material associated with vertical electronic transitions.

### 2.4 Experimental Methods

Ceria thin films were deposited using a Picosun R200 Advanced deposition atomic layer deposition (ALD) tool following the work of Vangelista et al.^67^ The [Ce(THD)_4_] (THD = 2,2,6,6-tetramethyl-3,5-heptanedione) precursor was purchased from EpiValence Ltd. and used as supplied. It was evaporated at a temperature of 195 °C with ozone used as the oxidizing agent. Both precursors were transported into the reactor chamber by an N_2_ carrier gas flow, at a deposition temperature of 250 °C. The conditions for the [Ce(THD)_4_] precursor were a carrier flow of 200 sccm, a pulse time of 2.0 s, and a purge time of 6.0 s, while for ozone were 50 sccm, 1.0 s, and 3.0 s with the ozone generator set to 50% power. The intermediate space flow was 150 sccm, and all other lines were set to 50 sccm. Depositions were performed between 300 and 1900 ALD cycles. The deposition was performed on both indium tin oxide (ITO)-coated glass (which was half-covered with a silicon coverslip to allow electrical connection to the ITO for XPS measurements) and silicon wafer (for thickness measurements) simultaneously.

Spectroscopic Ellipsometry (SE, Woollam M-2000 Test Base) was used to acquire spectra in the visible-UV range from 1.24 to 5.05 eV. The film thickness and refractive index of CeO_2_ on the silicon wafer were obtained by applying a Cauchy model for fitting ellipsometric data obtained at an angle of 65° between 450 and 1000 nm wavelengths using Complete-EASE software (J.A. Woollam Co., Inc.). Under these deposition conditions, the growth rate of CeO_2_ on silicon was approximately 0.22 Å/cycle, somewhat lower than the 0.32 Å/cycle found by Vangelista.^67^

X-ray diffraction (XRD) was obtained using a PANalytical X’Pert3 X-ray diffractometer using Cu Kα radiation (λ = 1.540598 Å), in which the operating voltage was 40.00 kV and the operating current was 40.00 mA. 2θ (deg) was set from 10 to 80°, the incident angle θ (deg) was fixed at 1°/step, the scanning rate was set as 1 s/step.

Atomic force microscopy (AFM) measurements were performed in tapping mode on a Keysight 5500 AFM system using SCOUT 70 AFM probes from NuNano Ltd. (spring constant 2 N m^–1^ with the resonant frequency of 70 kHz and tip radius of curvature <10 nm). AFM tips were oscillated at the first harmonic of their resonant frequency during imaging (∼400 kHz). For each sample, four images were collected, with each image being 5 μm in size. A first-order flattening process was applied to the image to remove surface tilt. No further image processing was performed. The root mean squared (R.M.S.) roughness was calculated using the WsXM image analysis software for each image. The average and standard deviation of the four roughness values for the individual images were calculated and used to measure the R.M.S. roughness and associated error for each sample. The images were collected at different locations across the sample surface to ensure a consistent measure of the surface roughness.

XPS and UPS were carried out using a Thermo NEXSA spectrometer. The instrument utilized a 72W monochromated Al K-Alpha X-ray source (*E* = 1486.6 eV) focused to a spot of 400 μm diameter at the sample surface for XPS and a cold cathode He discharge lamp for UPS. The He lamp can produce either He(I) or He(II) emissions (21.1 and 40.8 eV, respectively). In UPS, to allow measurement of the work function, the sample was held at a −9 V bias voltage relative to the spectrometer using a metal contact to the underlying ITO. It was confirmed that the sample was in good electrical contact with the stage by observing a 1 eV/V shift in the spectroscopic features as the applied bias was changed. In XPS, charging was compensated for by the use of a dual beam (electron and Ar^+^ ion) flood gun. The electron energy analyzer consisted of a double-focusing 180° hemisphere with a mean radius of 125 mm, operated in constant analyzer energy (CAE) mode, and a 128-channel position-sensitive detector. The pass energy was set to 200 eV for survey scans and 50 eV for high-resolution regions. The binding energy scale of the instrument is regularly calibrated using a three-point energy reference (Ag, Au, and Cu). Spectra were analyzed using the Thermo Avantage software. Samples were immobilized on conductive carbon tape for analysis. Stability was assessed by time-resolved measurements of the core lines; no changes were observed, indicating that beam damage was not detectable on the time scale of these experiments. Spectra were charge corrected to adventitious C 1s at 285.0 eV.

## 3 Results and Discussion

### 3.1 Relative and Absolute Band Edge Positions in Bulk Cerium Oxides

**Figure 1:**
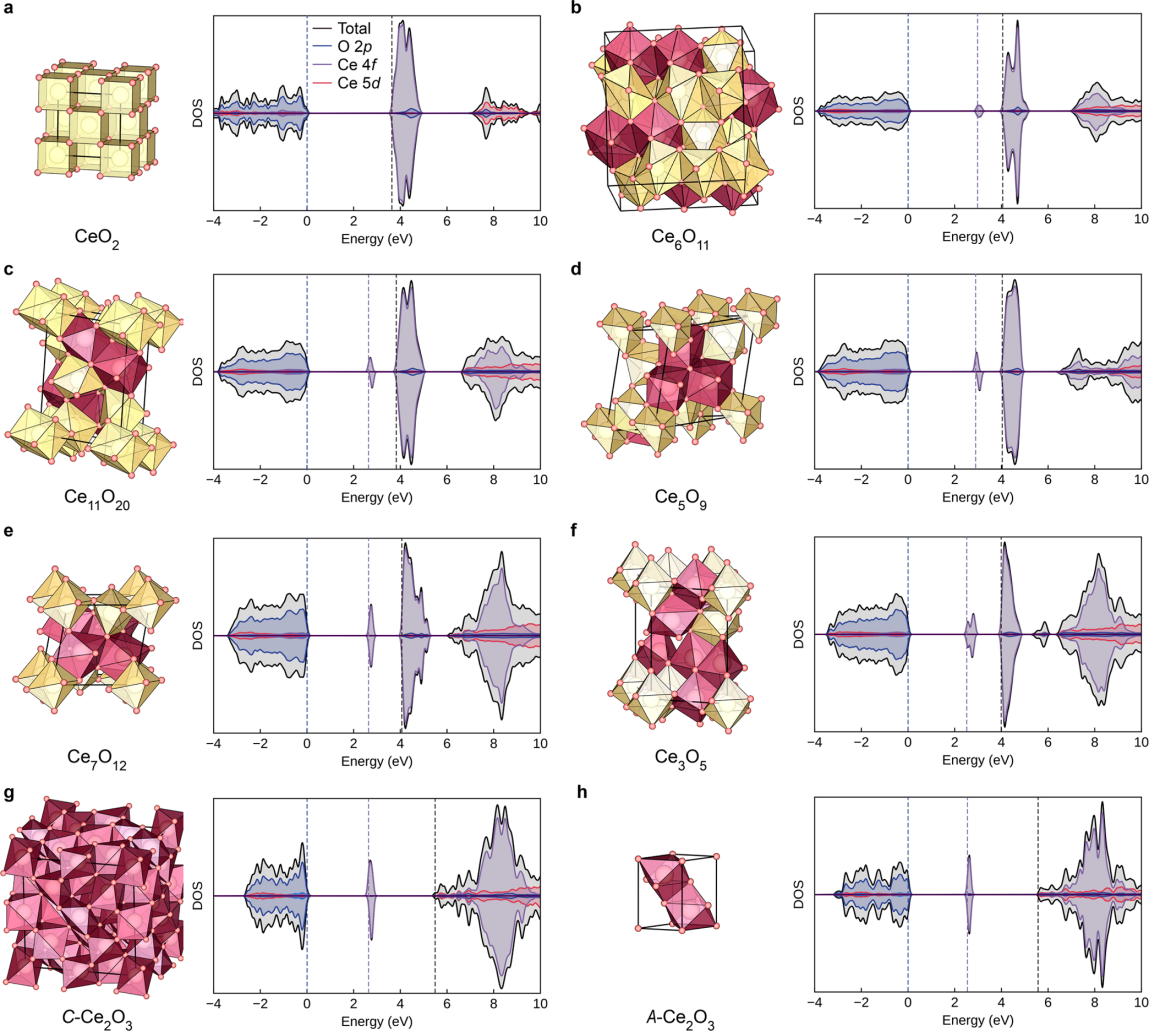
Optimized structures of CeO_2–*x*_ phases along with their electronic partial density of states (DOS) in the antiferromagnetic state calculated at the HSE06 level of theory by plane-wave DFT. Ce^4+^–O^2–^ and Ce^3+^–O^2–^ polyhedra are shown in yellow and magenta, respectively. The position of the highest occupied O_2p_ state in each system has been aligned to 0 eV. Blue, purple, and black dashed lines indicated the positions of the highest occupied O_2p_ and Ce_4f_ states and the lowest Ce_4f_ unoccupied state, respectively.

**Figure 2:**
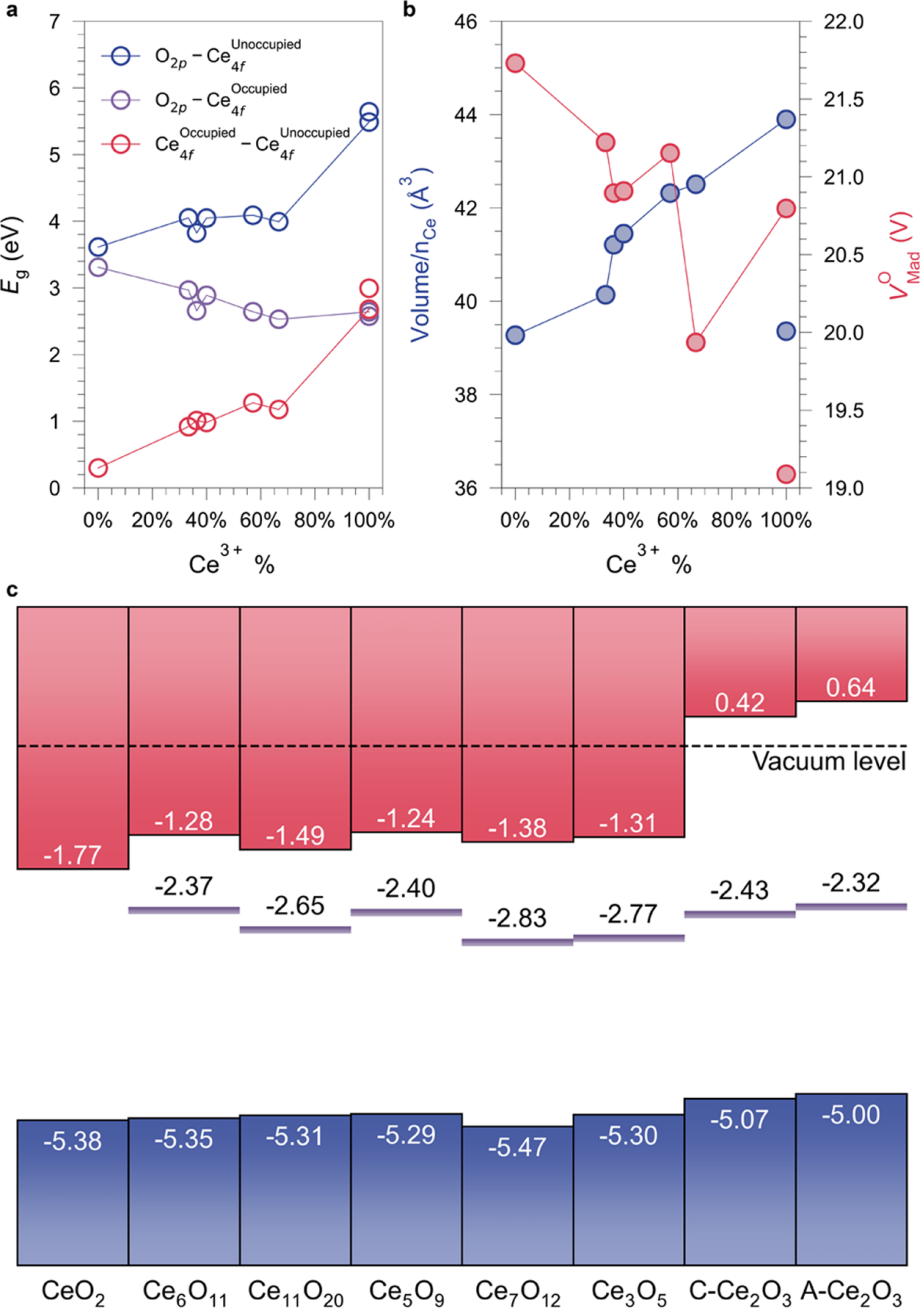
Electronic structure of ordered CeO_2–*x*_ (*x* = 0–0.5). (a) Variation in the energy gaps as a function of Ce^3+^ percentage calculated by plane-wave DFT using HSE06. For CeO_2_, the self-trapping energy of −0.30 eV for Ce^3+^ polarons in bulk CeO_2_ was adopted, as reported in ref (69). (b) Unit-cell volume per Ce atom and reduced Madelung potential on O sites (*V*_Mad_^O^) with the increased percentage of Ce^3+^ in CeO_2–*x*_. (c) Intrinsic band alignment of CeO_2–*x*_ phases excluding surface effects. The valence band edge positions were determined by the QM/MM approach at the BB1K level of theory, whereas the occupied Ce_4f_ (indicated by purple lines) and CBM positions were determined by adding the calculated band gaps using plane-wave DFT with the HSE06 functional.

The coexistence of Ce^4+^ and Ce^3+^ valences in cerium oxides results in a complex phase diagram between CeO_2_ and Ce_2_O_3_.^68^ We start by considering the band structure and bulk IP of several ordered cerium oxides. Plane-wave DFT calculations were performed at the HSE06 level of theory to obtain their crystal and electronic structures, as shown in Figure 1. CeO_2_ crystallizes in the cubic fluorite structure, where Ce^4+^ cations have a coordination number (CN) of 8 (Figure 1a). The VBM of CeO_2_ is predominantly composed of the O_2p_ states hybridized with some Ce orbitals of d and f character, while the CBM mainly originates from unoccupied Ce_4f_ states, resulting in an experimental optical band gap of 4 eV^48^ (3.61 eV by HSE06 calculation) between the occupied O_2p_ and unoccupied Ce_4f_ states. Under O-deficient conditions, CeO_2_ readily forms oxygen vacancies, with excess charge-compensating electrons localized on cations to form small polarons (Ce_Ce_^′^, or Ce^3+^). At the dilute limit, the small polaronic state in CeO_2_ lies about 0.4 eV below the unperturbed CBM, as confirmed by experiment and theory.^48,69^ In reduced CeO_2–*x*_ phases, the cation CN can be 6, 7, or 8, determined by the concentration and distribution of oxygen vacancies. From HSE06 DFT calculations, we observed an increased energy gap between the highest occupied and lowest unoccupied Ce_4f_ states correlating with the reduced oxygen stoichiometry (Figure 2a), from 0.4 eV of the polaronic state in CeO_2_ to 2.68 eV for C-Ce_2_O_3_ and 3.00 eV for A-Ce_2_O_3_, respectively. The calculated energy gap between the occupied O_2p_ and unoccupied Ce_4f_ states also widens from 3.61 eV in CeO_2_ to 5.49 eV in C-Ce_2_O_3_ and 5.64 eV in A-Ce_2_O_3_, respectively, due to the insertion of occupied Ce_4f_ states in between. In contrast, the calculated band gap between the O_2p_ and occupied Ce_4f_ states remains within a narrow range from 2.53 to 2.74 eV across all reduced phases. The same trends are also observed in PBE0 and PBE + *U* calculations, as shown in Table S1.

While plane-wave DFT calculations using effective core pseudopotentials provide reasonable estimates of the relative band edge positions, their absolute positions and alignment are unknown due to the lack of a common reference under periodic boundary conditions. As noted, the hybrid QM/MM embedded-cluster approach is the state-of-the-art theoretical model to obtain the absolute band edge positions in solids.^25,58,70^ The point charges associated with the MM regions, tailored to reproduce the long-range periodic atomic structure and electrostatic environment in ionic solids, offer an embedding potential for the QM cluster. The avoidance of periodic boundary conditions in our QM/MM models maintains access to the vacuum level, allowing for direct comparison among different materials under a common reference satisfying the tinfoil boundary conditions.^71,72^ With this methodology, we calculated the absolute valence band edge positions in reduced phases of ceria, which show effects only from the bulk lattice, excluding the impact from surfaces at this stage. As shown in Figure 2c, QM/MM calculations predict that the occupied states in bulk CeO_2–*x*_ phases are closely aligned. As CeO_2_ is reduced, the −5.38 eV O_2p_ band edge in CeO_2_ shifts slightly to approximately −5.0 eV in both Ce_2_O_3_ phases. The absolute positions of occupied Ce_4f_ states also show minor variations with stoichiometry. The small differences in energetics indicate that the bulk structure has a relatively small contribution to the pronounced variations in the IP and Φ of reduced ceria observed in the experiment,^23^ and surface effects should be taken into account for a full understanding.

The overall upward trend in VBM shifting with an increased extent of reduction in cerium oxides could be mainly attributed to modifications in local electrostatics. We employed shell-model interatomic potential techniques to calculate the on-site Madelung potential (*V*_Mad_) across various phases. In metal oxides, the Madelung potential on the O-site (*V*_Mad_^O^) is a key descriptor for determining the O_2p_ band edge positions.^25^ A higher *V*_Mad_^O^ implies a stronger binding of electrons to the anion site, typically leading to a higher IP (or a more negative VBM in the energy spectrum). Moreover, the reduction of CeO_2_ is accompanied by lattice expansion due to the formation of larger Ce^3+^ ions (Figure 2b). As the level of reduction increases, there is a declining trend in the lowest *V*_Mad_^O^ in the bulk lattice, driving the elevation of the VBM as predicted from our QM/MM calculations (Figure 2c). However, compared to the significant decrease in *V*_Mad_^O^, the VBM shows only minor shifts with some fluctuations. This phenomenon can be attributed to the evolving second electron affinity of oxygen in ceria during reduction, which is a variable known to be structure-dependent in metal oxides. Using our interatomic potential, we calculated the lattice energies for CeO_2_ (−107.50 eV) and A-Ce_2_O_3_ (−129.41 eV), along with the respective in-lattice second electron affinities of oxygen, employing the Born–Haber cycle as described in our previous study.^26^ The second electron affinity of oxygen is found to increase from 8.14 eV in CeO_2_ to 9.26 eV in A-Ce_2_O_3_, due to a change in the coordination environment. Therefore, the closely aligned VBM positions in bulk cerium oxides, as observed in hybrid QM/MM electronic structure calculations, stem from a balance between the reduced electrostatic Madelung potential and enhanced second electron affinity at oxygen sites on reduction.

### 3.2 Effects of Surface Reduction in O-poor Conditions

With a clear understanding of the bulk contributions to the absolute band edge positions in reduced ceria, we then considered surface effects. We combined several theoretical and experimental techniques to probe the impact of variable stoichiometry and surface chemistry on the IP and Φ of ceria. The effect of surface orientation has been demonstrated in our previous work.^25^ Here, our theoretical investigation centered on the variable surface chemistry on the most stable CeO_2_(111) surface. A key feature of reduced ceria is that the charge-compensating electrons (Ce^3+^ polarons) are not tightly trapped near the vacancy site.^26,69^ In bulk CeO_2_, excess electrons can localize within the first and second coordination shells of the vacancy site with similar stabilities, as shown by our previous QM/MM, Mott–Littleton, and other periodic DFT calculations.^26,73^ At the dilute limit, the formation energy of a charge-neutral oxygen vacancy in bulk CeO_2_ in O-rich conditions was calculated as 3.70–4.04 eV at the QM/MM PBE0 level theory (3.56–3.66 eV from Mott–Littleton calculations), with the variance arising from different Ce^3+^ locations around the V_O_ site.^26^ On CeO_2_(111), Sauer et al.^74^ also highlighted that subsurface vacancies can be more stable than surface vacancies, and the excess electrons may not be in close proximity to the vacancy sites. The formation energies of surface and subsurface vacancies were reported to be 3.00 and 2.77 eV, respectively, at the PBE0 level of theory calculated using slab models, which are ca. 1 eV lower than those in the bulk.^75^ Furthermore, the energy barriers for V_O_ migration (ca. 0.52 eV^26^) and Ce^3+^ polaron hopping (0.1–0.2 eV^69,76^) are also very low, with potential coupling mechanisms enhancing their surface mobility.^77^ These theoretical results suggest a preference for the segregation of vacancies and polarons close to surfaces, while the atomic configurations of CeO_2–*x*_ surfaces are expected to be very complex and dynamically evolving under operational conditions.

**Figure 3:**
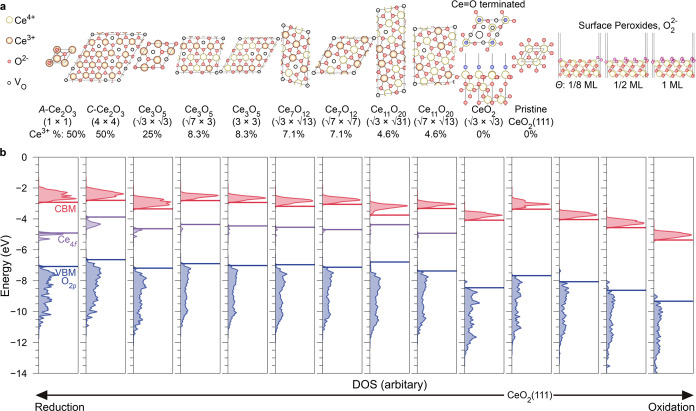
Variations of the density of states in (111)-terminated CeO_2_ in reducing and oxidizing environments. (a) Top views of various reconstructed patterns on reduced CeO_2–*x*_(111) surfaces in O-poor conditions and side views of different coverages of peroxide (O_2_^2–^) species formed in O-rich conditions. (b) Calculated density of states in the above models with respect to the vacuum level. The highest occupied O_2p_ states, Ce_4f_ states, and the lowest unoccupied states are marked in blue, purple, and red, respectively.

To assess the influence of surface reduction, we calculated the absolute band edge positions in various configurations of partially reduced CeO_2–*x*_(111) surfaces at the PBE0@PBE + *U* level of theory. Pristine CeO_2_(111) has a theoretical IP of 7.67 eV. In contrast to the bulk IP of 5.38 eV, the quadrupolar atomic arrangement elevates the *V*_Mad_^O^ and increases the surface IP by 2.3 eV, as demonstrated in our previous work.^25^ We first analyzed surface and subsurface vacancies in CeO_2_(111) using a *p*(4 × 4) slab model expansion, corresponding to a vacancy concentration of 1/16 in the surface or subsurface plane and 1/256 in the entire model. At this concentration, the formation of surface or subsurface vacancies reduces the IP from 7.67 eV for the pristine surface to 7.53 and 7.59 eV, respectively. Further reduction of CeO_2_(111) can form several ordered reconstruction patterns with various surface stoichiometries, as reported by Ganduglia-Pirovano et al.,^28,29^ many of which have been observed experimentally. Our atomistic models were constructed based on CeO_2_(111) with various concentrations and distributions of V_O_ and Ce^3+^ in the surface region (Figure 3a) while retaining the CeO_2_ lattice in the fixed bulk region, consistent with Ganduglia-Pirovano et al.^28,29^

Figure 3 illustrates how the absolute band edge positions in reduced or oxidized CeO_2–*x*_(111) are modulated by surface states. As the Ce^3+^ concentration exceeds 7.1% (equivalent to 3.55% of V_O_), the IP is already below 7 eV in most configurations. As a lower limit, surface reduction of CeO_2_(111) could decrease the IP by up to 1 eV, as seen in the C-Ce_2_O_3_ (4 × 4) reconstruction with 50% of Ce^3+^. The surface IP is also considerably affected by the vacancy and polaron distributions. For example, while the two reconstructed patterns under the Ce_11_O_20_ stoichiometry share the same defect concentration, the IP of the () type of reconstruction is 0.58 eV lower than that of the () type. This sensitivity to defect distribution can be seen in the A-Ce_2_O_3_ (1 × 1) and C-Ce_2_O_3_ (4 × 4) reconstructions, where the topmost four trilayers of atoms are reduced to two types of Ce_2_O_3_ phases, with 0.44 eV difference in their IPs. Recently, Grinter et al.^49^ discovered a novel Ce=O terminated  ×  reconstruction on CeO_2_(111) stabilized in O-deficient environments, which loses Ce and O stoichiometrically while forming weak Ce=O bonds on top of surface Ce, yielding an IP (8.47 eV) even higher than the pristine surface. Compared to the O_2p_ states, the position of the highest occupied Ce_4f_ state in reduced ceria is more sensitive to the reconstruction patterns, ranging from −3.88 eV (for C-Ce_2_O_3_ (4 × 4) reconstruction) to −5.48 eV (for a single subsurface vacancy).

From a crystallographic perspective, the lattices of ordered reduced phases can be converted to the cubic fluorite unit-cell basis. We also calculated the IPs of ordered reduced phases under the fluorite(111) surface termination to be compared with pure and partially reduced CeO_2_(111). The calculated IPs from the O_2p_ states of Ce_6_O_11_(001), Ce_11_O_20_(201̅), Ce_7_O_12_(111), C-Ce_2_O_3_(111), and A-Ce_2_O_3_(001) are 7.31, 6.28, 6.22, 6.53, and 7.19 eV, respectively. Ionization from the highest occupied Ce_4f_ states requires 5.11, 4.22, 4.46, 4.59, and 4.87 eV, respectively. Compared to the pure bulk contributions shown in Figure 2c, a more significant difference in the absolute band edge positions is observed when surface effects are included. This change can be attributed to a balance of several factors, including surface polarization, atomic relaxation, and distribution of vacancies and Ce^3+^ near the surface, with their influence varying across different systems.

**Figure 4:**
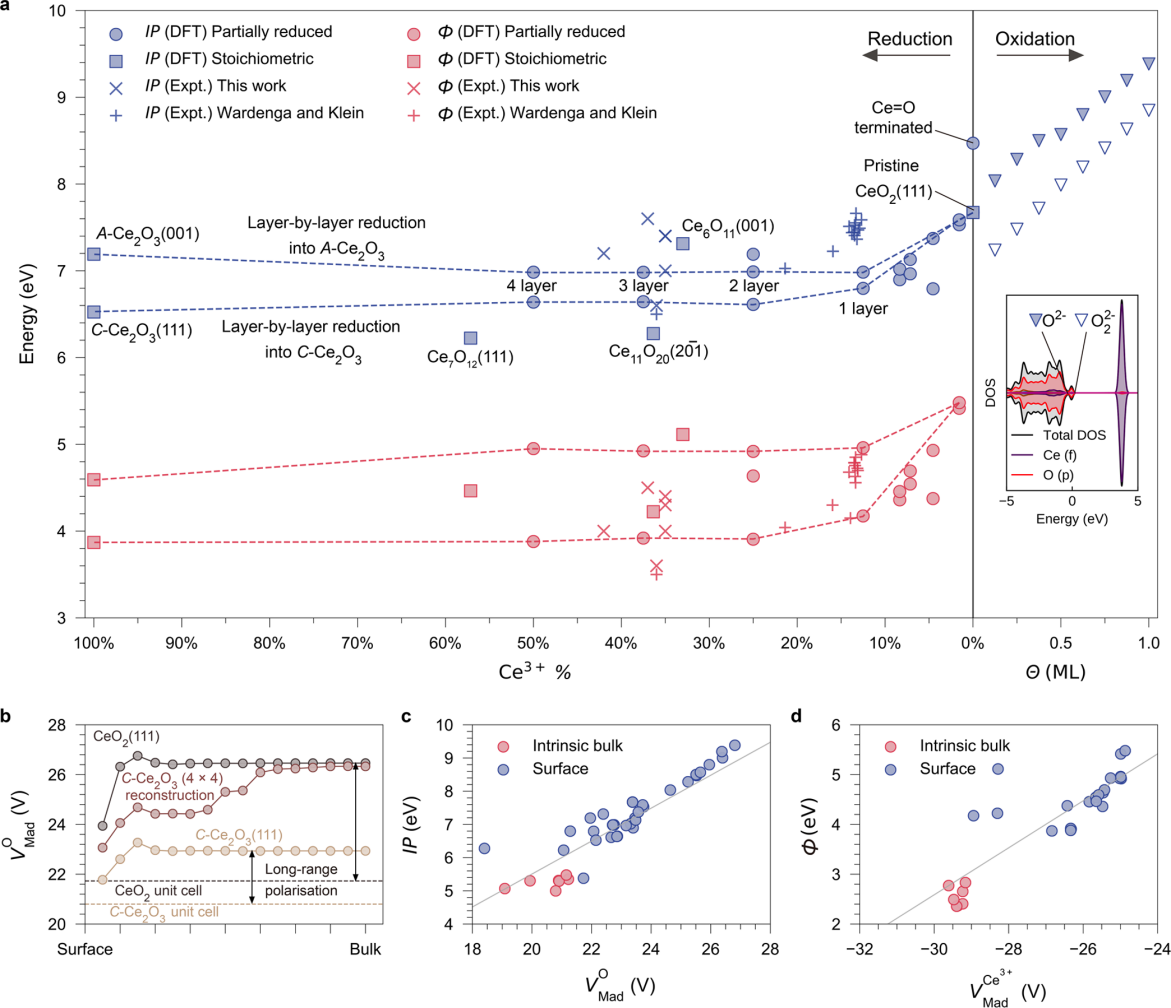
Environmental modulation of IP and Φ of cerium oxides and its relevance to electrostatics. (a) Variation of IP and Φ in (111)-terminated CeO_2_ in different reduction and oxidation environments from DFT calculations and experimental measurements in this work as well as by Wardenga and Klein.^23^ The inset is the DOS of the 1-ML-peroxide-covered (111) surface, showing a split valence band due to lattice O^2–^ (filled triangles) and surface O_2_^2–^ (empty triangles). (b) Layer-by-layer distribution of *V*_Mad_^O^ penetrating from the surface layer to the bulk region under pristine CeO_2_(111), partially reduced C-Ce_2_O_3_ (4 × 4) reconstruction on CeO_2_(111), and fully reduced C-Ce_2_O_3_(111). Dashed lines indicate the *V*_Mad_^O^ calculated in periodic unit cells of CeO_2_ and C-Ce_2_O_3_, respectively, which are the pure bulk contributions to the electrostatics that exclude surface effects. The double arrows highlight the differences in *V*_Mad_^O^ calculated in periodic unit cells and in the bulk region under the (111) surface of CeO_2_ and C-Ce_2_O_3_, which originate from long-range surface polarization. (c) Relationship between the IP of reduced ceria with the lowest *V*_Mad_^O^. (d) Relationship between the Φ of reduced ceria with the lowest *V*_Mad_^Ce^3+^^.

Figure 4a summarizes the environmental effects on the IP and Φ in cerium oxides, as derived from our theoretical calculations and experimental measurements, together with previous measurements by Wardenga and Klein.^23^ Generally, the IP of cerium oxides tends to decrease with an increasing degree of reduction. However, the IP reduction in CeO_2–*x*_(111) does not correlate linearly with defect concentration. There is a sharp decline in the calculated IP during the initial surface reduction when the Ce^3+^ proportion is below 20%, which subsequently stabilizes around 7 eV. We also simulated the layer-by-layer surface reduction processes through a sequential modification of surface atomic configurations by A-type and C-type Ce_2_O_3_ on the CeO_2_(111) substrate. As highlighted by the dashed lines in Figure 4a, the resulting IPs show negligible variations after the formation of the uppermost reduced layer, indicating that the topmost surface layer has a dominant effect on the absolute energy levels in metal oxides. This result aligns with a previous study by Butler et al.,^78^ which demonstrated that a capping monolayer (ML) on SnO_2_(100) with different oxides (such as SiO_2_, TiO_2_, and PbO_2_) is capable of modulating the IP either upward or downward. The experimental IP measurements on thin-film samples also showed a notable IP uncertainty in reduced ceria for a given stoichiometry, which is probably a consequence of more disordered defect configurations. Moreover, the perturbation of VBM in reduced ceria invariably influences the absolute position of the Fermi level. During the reduction of ceria, the absolute Fermi level is not only modulated by the concentration of charge carriers within the band gap but also shifts based on VBM movement, leading to more pronounced fluctuations in Φ, as seen in Figure 4a.

The IP differences among reduced ceria under the fluorite (111) orientation, although already pronounced, are not as large as the discrepancy caused by surface orientations in stoichiometric CeO_2_, which can span several eV, as shown in our previous work.^25^ For example, the IP of the nonpolar CeO_2_(110) surface is only 6.11 eV, much lower than the quadrupolar (111) surface of 7.67 eV. The IP of the polar (100) surface can vary from 4.21 to 8.20 eV due to different types of reconstruction and terminating ions. These discrepancies caused by orientation-dependent stacking sequences are expected to be further enhanced by the variable surface chemistry in realistic conditions. In this work, we focused only on the reduction of the most stable (111) surface of CeO_2_, and surface reduction is expected to have a similar effect on other ceria surfaces.

### 3.3 Effects of Surface Oxidation and Formation of Peroxides in O-rich Conditions

In O-rich experimental conditions, as reported by Wardenga and Klein,^23^ both the IP and Φ of their samples increased significantly by 1–2 eV after the O_2_ plasma treatment, without an observable reduction in the Ce^3+^ concentration. We attribute this phenomenon to the formation of surface peroxide (O_2_^2–^) species. The oxidation of reduced ceria surfaces not only heals the near-surface vacancies upon adsorption but also can lead to the formation of peroxide species (Figure 3), as confirmed by a recent study of Cuenya et al.^34^ on O_2_ plasma-treated CeO_2_ samples using a combination of XPS, Raman spectroscopy, and DFT calculations.

We conducted plane-wave DFT calculations based on the CeO_2_(111) slab model with different coverages of O_2_^2–^ species. As can be seen in the small prepeak in the DOS plot of Figure 4, the energy level of the surface O_2_^2–^ species splits from the filled O_2p_ band of lattice oxygen and positions itself marginally above the original band edge. If we separately consider the energy levels of O^2–^ (marked in hollow triangles) and O_2_^2–^ (marked in solid triangles), the formation of surface peroxide species results in a downward shift of the filled O_2p_ bands of the lattice oxygen. As a result, with the increase in the surface coverage of O_2_^2–^, there is an ascending trend in the IP, reaching up to 9.38 eV at a coverage of 1 ML. The Fermi level is also expected to shift alongside the VBM and result in a higher Φ in oxidative environments. These results are consistent with the previous observation by Wardenga and Klein on plasma-treated (111)-terminated thin-film samples with a notably high IP of 8.6 eV and a Φ of 5.8 eV.^23^

### 3.4 Experimental Characterization of ALD-Deposited CeO_2–*x*_ Thin Films

**Figure 5:**
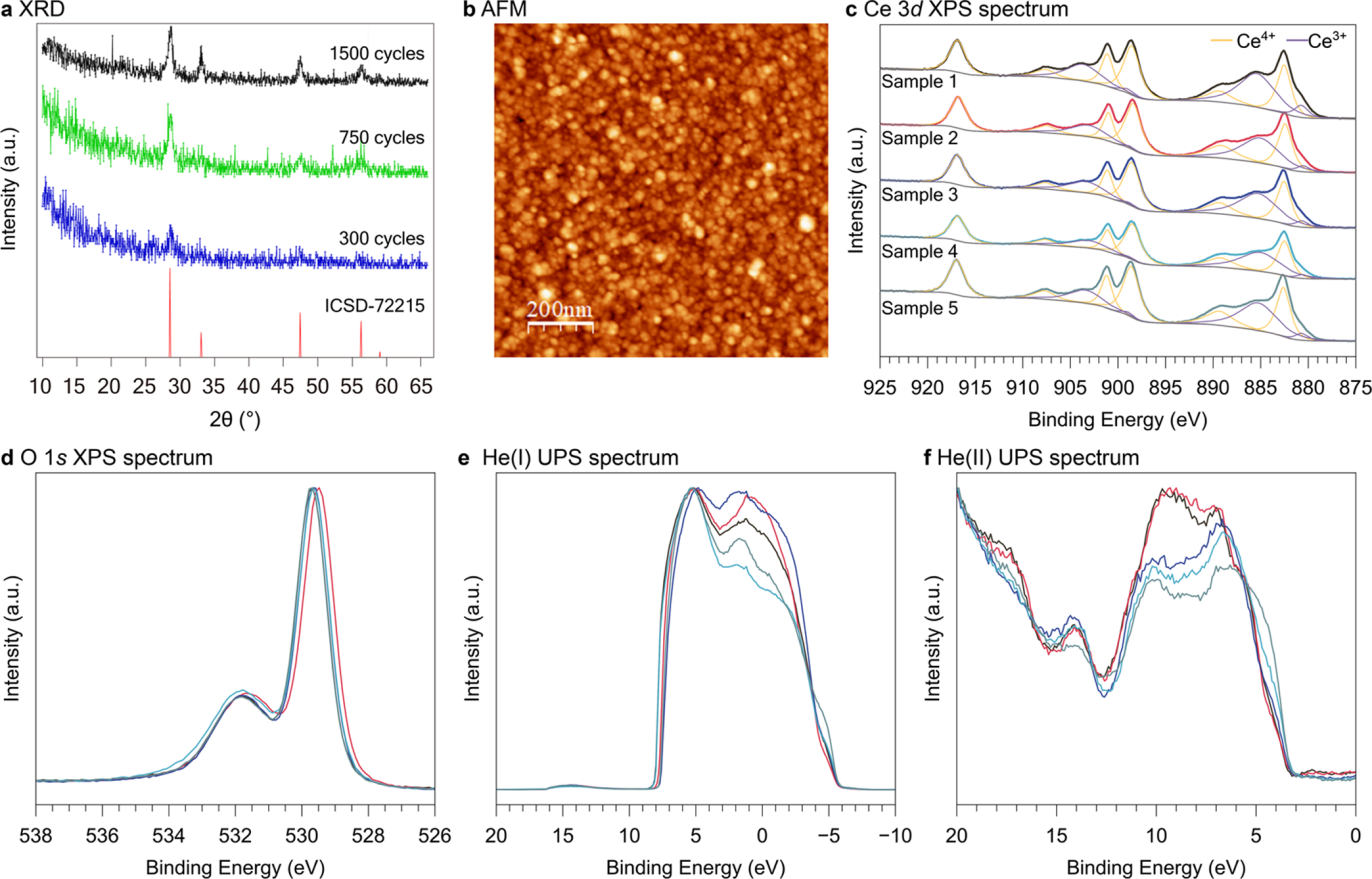
Experimental characterizations of CeO_2–*x*_ thin films prepared by ALD. (a) XRD analysis on ceria thin films prepared by different numbers of ALD cycles. (b) Surface topography of ceria ultrathin film characterized by AFM. (c) The Ce 3d XPS spectrum. (d) The O 1s XPS spectrum. (e) The He(I) UPS spectrum at −9 V sample bias. (f) The He(II) UPS spectrum.

We prepared CeO_2–*x*_ thin films with a thickness of approximately 20 nm using ALD (900 cycles) and characterized their electronic properties using a combination of XPS and UPS to reveal the variations of IP and Φ of reduced ceria under different environments (Table 1). XRD analysis verified the expected peak positions from CeO_2_ (Figure 5a), aligning with the ICSD database entry ICSD-72155. A progressive enhancement in diffraction intensity was observed with an increase in ALD cycles, with the principle CeO_2_ peaks becoming pronounced by 750 cycles. AFM characterization revealed the polycrystalline nature of the thin-film samples, with observed grain sizes ranging from 10 to 50 nm and rough surface topographies (Figure 5b). The oxidation states of Ce in the 20 nm-thick films were probed using the Ce 3d XPS spectra to evaluate the degree of reduction, as shown in Figure 5c. While absolute binding energies have a degree of uncertainty due to the lack of a reliable reference point, the oxidation state of Ce can be determined based on spectral shape rather than precise binding energy.^79,80^ XPS analysis revealed a significant presence of Ce^3+^, suggesting the ease of forming oxygen vacancies in ALD-derived thin films. The as-deposited Sample 1 has a substantial 42% percentage of Ce^3+^ ions, as inferred from the pronounced binding energy shoulders at 885.7 and 903.8 eV in its XPS spectra. Other samples, subject to postannealing or O_2_-plasma treatment, all showed consistent Ce^3+^ percentages in the range of 35–37%, irrespective of the annealing temperature or environmental conditions.

**Table 1:** Thickness, Ce^3+^ Percentage, IP, and Φ for ALD-derived CeO_2–*x*_ Samples, measured by XPS and UPS

sample ID	sample	thickness (nm)	Ce^3+^ (%)	IP (eV)	Φ (eV)	IP-Φ (eV)
sample 1	as-deposited	23	42	7.2	4.0	3.2
sample 2	O_2_ plasma@250 °C	19	35	7.4	4.3	3.1
sample 3	O_2_ annealed@350 °C	18	37	7.6	4.5	3.1
sample 4	Air annealed@250 °C	19	35	7.0	4.0	3.0
sample 5	O_2_ annealed@250 °C	21	35	7.4	4.4	3.0

Further analysis of the O 1s spectra revealed a consistent multipeak profile across all samples (Figure 5d). Deconvolution of these spectra showed the presence of three types of oxygen species (Figure S1), indicating a complex surface chemistry. The main peak, observed at approximately 529.3 eV, corresponds to the lattice oxygen in ceria, comprising 70.5% of the total oxygen atoms. The two small peaks, observed at approximately 531.0 and 532.0 eV, account for 16.4 and 13.1% of the oxygen composition, respectively. The smallest peak at 532.0 eV is typically assigned to carbonate species.^34^ The 531.0 eV peak in the O 1s spectrum could be assigned to oxygen bound to carbon in a surface adventitious carbon layer. Moreover, the C 1s spectrum shown in Figure S2 identified four carbon environments. The main peak located at 285 eV is associated with adventitious carbon, a consequence of air exposure. The other three peaks at 287.2, 289.1, and 290.9 eV correspond to C–O, C=O, and carbonate (CO_3_^2–^) species, respectively.

The detection of surface carbonate species implies a potential deviation from the ideal CeO_2–*x*_ composition. While XPS does not yield accurate concentrations of species residing only on the surface, the deconvoluted peak areas in the O 1s spectra suggest an approximate ratio of 1:13 between CO_3_^2–^ and lattice O^2–^. We performed DFT calculations to evaluate the effects of surface carbonates on the IPs of ceria thin films. We absorbed a CO_2_ molecule onto the CeO_2_(111) surface slab, resulting in the preferential formation of monodentate carbonate configurations (Figure S3a), consistent with previous research.^81^ Carbonate coverages of 1/16 ML and 1/8 ML were found to increase the surface IP by 0.40 and 0.81 eV, respectively, due to an increased surface dipole. The existence of surface oxygen vacancies and Ce^3+^ can facilitate CO_2_ adsorption and the formation of carbonate species (Figure S3b,c). These defects serve to counterbalance the surface dipole induced by carbonate species, thereby resulting in a more modest increase in surface IP by 0.22 and 0.48 eV for 1/16 ML and 1/8 ML of coverages, respectively. Given the defective nature of the ALD-derived thin films, this model could be more consistent with experimental conditions. These calculations suggest that the presence of surface carbonate species in the ALD-deposited ceria thin films could induce a slight increase in the measured IP by up to 0.48 eV compared to pure cerium oxides. However, the consistent shapes of the O 1s spectra across the samples suggest a similar concentration of carbonates, presumably leading to a uniform influence on their surface potentials.

The IP and Φ of the CeO_2–*x*_ thin films were determined by UPS with He(II) and He(I), respectively, as the excitation sources (Figure 5e,f). The measurements on 20 nm-thick CeO_2–*x*_ films confirmed that both IP and Φ increased in oxidizing environments. The IP of the as-deposited sample 1 was measured as 7.2 eV, compared with 7.4 eV of sample 5 with postannealing treatment in O_2_ at 250 °C and further with sample 3 of 7.6 eV annealed at 350 °C. Meanwhile, the Φ also rose from the as-deposited 4.0 eV to 4.3–4.4 eV at 250 °C and 4.5 eV at 350 °C. However, the effect of O_2_ plasma (sample 2) is less pronounced than the previous report by Wardenga and Klein.^23^ At 250 °C, the O_2_ plasma effect is similar to postannealing in O_2_. This discrepancy could stem from the insufficient operational temperature to overcome the oxygen migration barrier to heal the oxygen vacancies, as reflected in the unchanged 35% of Ce^3+^ in plasma-treated sample 2, compared with the sample with 13% of Ce^3+^ derived at 700 K by Wardenga and Klein.^23^ In comparison, air annealing (sample 4) resulted in a lower IP of 7.0 eV, where the oxygen partial pressure is lower. The IP of our CeO_2–*x*_ samples exhibited less variability compared to those by Wardenga and Klein,^23^ who have studied a broader temperature range up to 700 °C. However, the general trend observed in our study is consistent with previous work and is comparable with our theoretical predictions.

Despite similar Ce^3+^ percentages in samples 2–5, the measured IP ranged from 7.0 to 7.6 eV, and Φ varied from 4.0 to 4.5 eV, highlighting the critical roles of defect distribution and surface conditions in affecting the absolute energy levels, in alignment with our theoretical predictions. Nevertheless, the energy differences between IP and Φ maintained at 3.0–3.2 eV across all samples, reaffirming a synchronized environmental modulation of the highest occupied O_2p_ and Ce_4f_ band edges, as predicted by our theoretical calculations. These findings emphasize the profound sensitivity of IP and Φ to environmental conditions and to the postdeposition treatment and defect configurations in CeO_2–*x*_, which could be strategically exploited to tailor the band edges in metal oxides for applications in photocatalysis and electronic devices.

### 3.5 Critical Role of Electrostatics

In metal oxides, near-surface ions experience distinct electrostatic environments compared to their bulk counterparts due to their reduced coordination numbers and surface electronic polarization. The correlation between the *V*_Mad_^O^ and IP proposed in our previous work^25^ can be further extended to CeO_2–*x*_ systems with variable surface chemistry. In Figure 4b, we plot the layer-by-layer distribution of *V*_Mad_^O^ as penetrating from the surface into the bulk in three models: pristine CeO_2_(111), partially reduced C-Ce_2_O_3_ (4 × 4) reconstruction with 50% of Ce^3+^ and fully reduced C-Ce_2_O_3_(111) surfaces. The IPs of the three surface models were calculated as 7.67, 6.64, and 6.53 eV by DFT, respectively. In all cases, there are significant fluctuations of *V*_Mad_^O^ near the surface region, which converges rapidly as it extends into the bulk region. The quadrupolar pattern of stacking sequence under the (111) surface significantly increases *V*_Mad_^O^ from the surface to the bulk, manifested as a long-range effect,^25^ as seen in the elevated *V*_Mad_^O^ (26.46 eV) in the bulk region under the (111) surface compared with 21.73 eV (dark dashed line in Figure 4b) calculated in the intrinsic CeO_2_ unit cell (excluding surface effects). This phenomenon was attributed to polarization associated with surface formation, where surface orientation, electronic redistribution, and atomic relaxation contribute to modifications in the Madelung potentials from the surface to the bulk region of metal oxides.^25^ In comparison, fully reduced C-Ce_2_O_3_(111) shows both a lower bulk electrostatic contribution (20.80 V) due to the reduced charge and enlarged size of cations and a weaker long-range polarization resulting from the decreased dielectric constants. The partially reduced C-Ce_2_O_3_ (4 × 4) reconstruction model combines the features of both surfaces: the capping C-Ce_2_O_3_(111) layers retain a comparatively low *V*_Mad_^O^, which increases rapidly at the Ce_2_O_3_/CeO_2_ interface and eventually approaches the values in the bulk region of CeO_2_(111). This variation in the electrostatic environment results in a decrease in surface IP with an increased degree of reduction.

Figure 4c shows a linear relationship between the electrostatically derived *V*_Mad_^O^ and DFT-calculated IP, covering all studied QM/MM bulk and surface slab models. A higher *V*_Mad_^O^ indicates an enhanced electron binding at anionic sites, suggesting a higher energy required to extract an O_2p_ electron from the VBM (IP). A similar relationship was found between *V*_Mad_^Ce^3+^^ and Φ in Figure 4d. A more negative *V*_Mad_^Ce^3+^^ on Ce^3+^ implies a stronger electrostatic attraction with the surrounding anions, and the Ce^3+^ cations require lower energy to ionize a Ce_4f_ electron to the vacuum (Φ). However, the trend is not as pronounced as the IP-V_Mad_^O^ relationship due to the variability of Fermi level shifts within the band gap. The environmental effects on the lattice structure, including oxygen vacancy formation, electron localization, surface absorption, surface reconstruction, and possible phase transformation, collectively result in modifications in both local and long-range electrostatic environments, further reflected in variations in absolute energy states.

These analyses confirmed the critical role of in-lattice electrostatics in metal oxides in determining their absolute band edge positions. However, the Madelung potential, although highly correlated with the calculated IP and Φ, is not the only determinant. Factors that affect the second electronic affinity of oxygen in the lattice, such as the lattice structure and cationic properties, also contribute. As a result, the linear relationship between IP and *V*_Mad_^O^ is more significant for describing the same material under different surface terminations compared to its description across various metal oxides.^25^

### 3.6 Effects of Surface Adsorbates and Impurities

**Figure 6:**
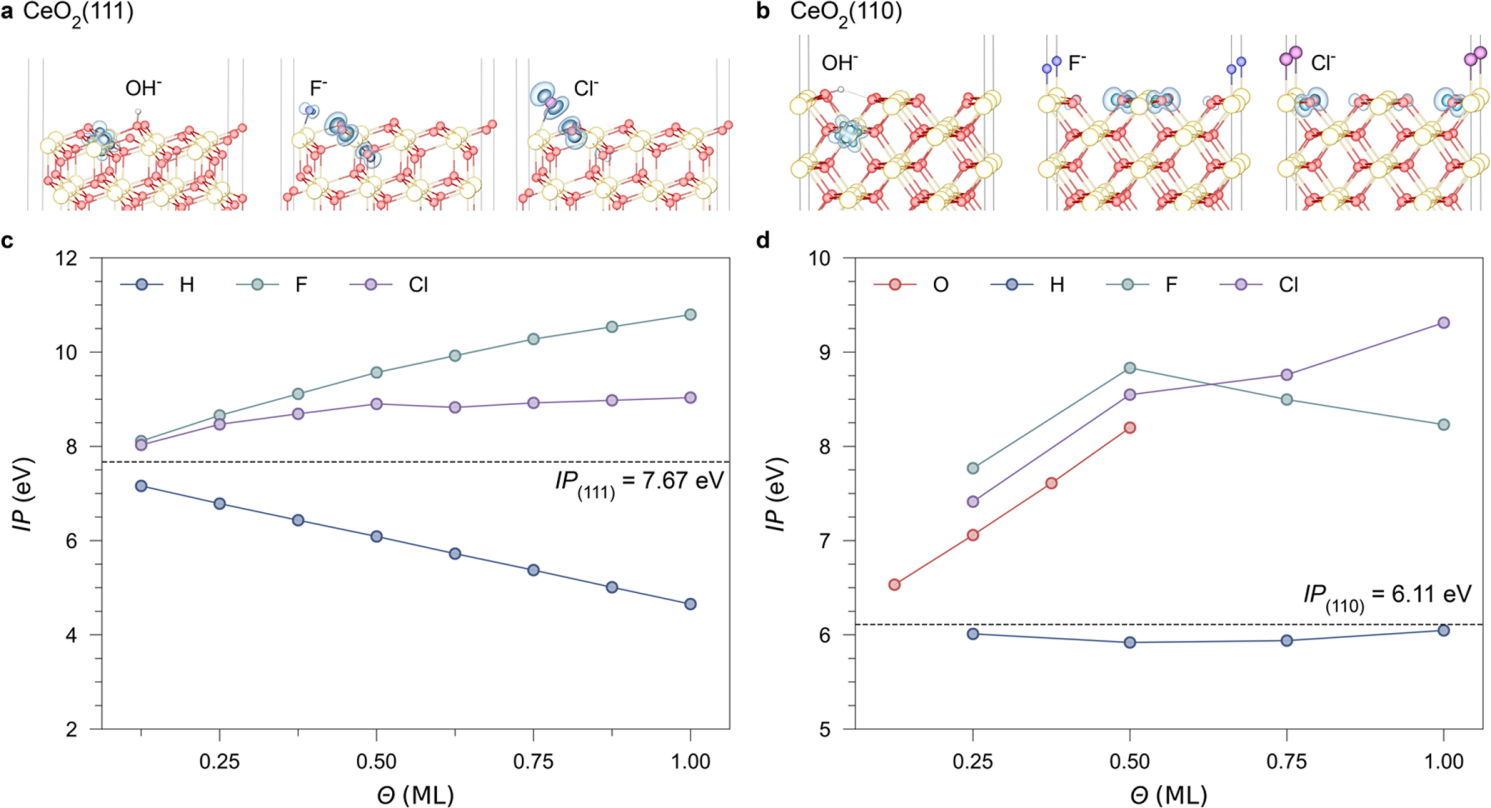
Effects of surface adsorbates and impurities on the IPs of CeO_2_ surfaces. Configurations of surface adsorbates on (a) CeO_2_(111) and (b) CeO_2_(110) are shown along with the variation of the IPs of (c) CeO_2_(111) and (d) CeO_2_(110) with different adsorbate coverages. Dashed lines indicate the reference IPs of pristine surfaces.

The critical role of the topmost surface layer in affecting the absolute energy levels of ceria stimulates further investigations of the impact of surface adsorbates and impurities. Hydrogen, a ubiquitous adsorbate on oxide surfaces from various sources,^82,83^ readily adsorbs on ceria surfaces. Under catalytic conditions, ceria surfaces can be fully hydroxylated, which significantly modifies their physical and chemical properties.^84,85^ The hydrogenation at surface O sites is charge-compensated by Ce^3+^ polarons (Figure 6a). The resultant surface hydroxyl groups are dipolar, creating an electrostatic field directed from the positive hydrogen centers toward the bulk. Such an electrostatic field has a long-range effect on bulk electrostatic potentials, on CeO_2_(111), promoting the ionization of electrons from the surface, as shown in the decreasing IP with increased surface coverage by OH^–^ groups (Figure 6c). In contrast, the IP of CeO_2_(110) remains nearly unaffected by hydroxyl groups (Figure 6d). The hydrogen adsorbed on CeO_2_(110) forms hydrogen bonds with other surface anions (Figure 6b). The resulting O–H bond and its induced electrostatic field are nearly parallel to the surface, minimizing the surface dipole, thereby having little effect on the bulk electrostatic environment. This comparison highlights the role of molecular orientation in adsorption on metal oxide surfaces.

Fluoride, a common contaminant in ceria samples, usually segregates on the surface while being below the XPS detection threshold.^86,87^ On CeO_2_(111), the formation of surface-adsorbed F^–^ species is compensated by holes, which mainly localize on near-surface O sites (Figure 6a,b). The high electronegativity of adsorbed F^–^ species on CeO_2_ surfaces induces a substantial surface dipole, whose direction is opposite to that induced by OH^–^, which increases the IP and Φ significantly with the increased coverage. A similar situation is seen in surface chloride (Cl^–^) species, while its effect on valence-band shifting is weaker than that of F^–^ due to its lower electronegativity (Figure 6c,d). Finally, like CeO_2_(111), surface peroxide species formed on CeO_2_(110) also increase the IP from 6.11 to 8.30 eV at a coverage of 0.5 ML.

Figure S4 shows layer-by-layer DOS of CeO_2_(111) and CeO_2_(110) with different types of surface adsorbates at a common coverage of 1 ML. Beyond influencing the conduction and valence band edges, surface species also affect the core-level positions and the associated band bending near the surface. While all energy bands shift in a consistent direction in response to the adsorbate-induced surface dipole, the direction and extent of the shifts vary across bands.

Overall, our results demonstrate that the IP and Φ of a metal oxide can be tuned extrinsically over a very large range by defect engineering, as well as by the type, coverage, and orientation of absorbed species on the surface. The growth conditions and surface treatment of a metal oxide have a significant impact on its physicochemical properties, thereby affecting the absolute band edge positions. Moreover, our study provides an explanation for the observed discrepancies in experimental measurements of IP, as well as the debate regarding the band alignment mechanisms. A typical case is the mixed rutile and anatase TiO_2_ phase, where experimental measurements and theoretical calculations have yielded several interpretations of the band alignment and charge transfer mechanisms.^58,88^ These discrepancies can be attributed to both intrinsic factors, such as surface orientations,^25^ and extrinsic factors, such as environmental conditions, which collectively affect the absolute band edges and their alignment. A recent theoretical study further showed that the absolute energy levels in TiO_2_ nanoparticles can be modified intrinsically by size and extrinsically by hydroxylation upon interaction with aqueous environments.^89^ Finally, in photocatalytic water splitting, the positions of the band edges of metal oxide catalysts are expected to evolve relative to the redox potentials of water due to the variation of surface species. Our theoretical findings highlight further the importance of *in situ* characterization techniques for capturing the electronic structure of photocatalysts in operating conditions.^90^ For example, Li et al.^91^ recently found that BiVO_4_ photoanodes showed different flat band potentials and magnitudes of surface band bending under electrochemical and photoelectrochemical water oxidation conditions, resulting in differences in hole generation mechanisms.

## 4 Conclusions

This work has illuminated the complex relationship between environmental conditions and the variable surface potentials of ceria. The combination of several theoretical and experimental techniques has shown a decreasing trend in both IP and Φ under oxygen-deficient conditions, which conversely increases in oxygen-rich environments owing to the formation of surface peroxides. Defect distributions were found to have a profound impact on the electronic properties of ceria, which can lead to 1 eV fluctuations in the IP and Φ despite a similar Ce^3+^ concentration. Surface adsorbates and contaminants can further amplify these variabilities, depending on their coverage, electronegativity, and orientation. The value of Φ not only changes within the band gap, which is determined by charge carrier concentrations, but also shifts concurrently with the absolute position of VBM, leading to greater variability than the IP. DFT calculations on embedded-cluster and periodic slab models allowed us to separate the individual contributions from bulk and surface to the observed energy-level shifting and to determine the critical effects of the topmost surface layer. Complementary electrostatic analyses underscore the critical role of on-site Madelung potentials in determining absolute energy levels. Our work provides a systematic understanding of the factors that govern the electronic properties of metal oxides, thereby paving the way for the rational design and engineering of oxide materials with tailored functionalities for catalytic and energy conversion applications.

## Supporting Information Available

Structures and properties of reduced CeO_2–*x*_ phases; interatomic potential parameters for reduced ceria and its performance; computational details of hybrid QM/MM calculations; deconvolution of the XPS spectra; and effects of surface adsorbates on absolute energy levels (PDF)
