# Supplementary material for: Environment-Driven Variability in Absolute Band Edge Positions and Work Functions of Reduced Ceria
Source: J Am Chem Soc. 2024 Jun 5;146(24):16814–29. doi: 10.1021/jacs.4c05053 (PMC11191696; doi:10.1021/jacs.4c05053)
Supplement: Supplementary file 1 — ja4c05053_si_001.pdf [file ja4c05053_si_001.pdf]

Supporting Information for

Environment-driven Variability in Absolute Band Edge Positions and Work Functions of Reduced Ceria

Xingfan Zhang[1, *], Christopher Blackman[2], Robert G. Palgrave[2], Sobia Ashraf[2], Avishek Dey[2], Matthew Oliver Blunt[2], Xu Zhang[1, 6], Taifeng Liu[1, 7], Shijia Sun[1], Lei Zhu[1], Jingcheng Guan[1], You Lu[3], Thomas W. Keal[3], John Buckeridge[4], C. Richard A. Catlow[1, 5, *], and Alexey A. Sokol[1, *]

1 Kathleen Lonsdale Materials Chemistry, Department of Chemistry, University College London, London WC1H 0AJ, United Kingdom.

2 Department of Chemistry, University College London, Christopher Ingold Building, 20 Gordon Street, London WC1H 0AJ, United Kingdom.

3 Scientific Computing Department, STFC Daresbury Laboratory, Warrington, Cheshire WA4 4AD, United Kingdom.

4 School of Engineering, London South Bank University, London SE1 OAA, United Kingdom.

5 School of Chemistry, Cardiff University, Park Place, Cardiff CF10 1AT, United Kingdom.

6 School of Chemical Engineering and Technology, Tianjin University, Tianjin 300350, P.R. China.

7 National & Local Joint Engineering Research Center for Applied Technology of Hybrid Nanomaterials, Henan University, Kaifeng 475004, China.

Corresponding authors:

* xingfan.zhang.20@ucl.ac.uk; c.r.a.catlow@ucl.ac.uk; a.sokol@ucl.ac.uk

Table S1. Lattice parameters and band gaps of CeO_2 and its ordered reduced phases calculated by plane-wave DFT at different levels of theories and by shell-model interatomic potentials, compared with available experimental measurements. The conduction band minimum (CBM) in reduced $CeO_{2\text{-}x}$ is made of a combination of 4*f* and 5*d* empty states. $\Delta E_{\text{AFM-FM}}$ represents the calculated energy difference per unit cell between the antiferromagnetic and ferromagnetic spin states.

	Source	*a* (Å)	*b* (Å)	*c* (Å)	*α* (°)	*β* (°)	*γ* (°)		$E_g^{2p\text{-}4f}$ (eV)	$E_g^{4f\text{-}5d}$ (eV)	$E_g^{2p\text{-}5d}$ (eV)
CeO_2	PBE+U	5.490	5.490	5.490	90.0	90.0	90.0		2.31	2.12	5.28
	PBE0	5.397	5.397	5.397	90.0	90.0	90.0		4.38	2.19	7.75
	HSE06	5.396	5.396	5.396	90.0	90.0	90.0		3.64	2.22	7.03
	SM	5.395	5.395	5.395	90.0	90.0	90.0				
	Expt.	5.395[a]	5.395	5.395	90.0	90.0	90.0		4.0[c]		6-8[b]
	Source	*a* (Å)	*b* (Å)	*c* (Å)	*α* (°)	*β* (°)	*γ* (°)	$\Delta E_{\text{AFM-FM}}$ (meV)	$E_g^{2p\text{-}4f}$ (eV)	$E_g^{4f\text{-CBM}}$ (eV)	$E_g^{2p\text{-CBM}}$ (eV)
A-type Ce_2O_3	PBE+U	3.920	3.919	6.174	90.0	90.0	120.0	-1.71	1.19	2.72	4.12
	PBE0	3.864	3.864	6.088	90.0	90.0	120.0	-3.74	2.55	3.74	6.37
	HSE06	3.864	3.864	6.090	90.0	90.0	120.0	-3.69	2.58	3.00	5.64
	SM	3.851	3.851	6.085	90.0	90.0	120.0				
	Expt.	3.882[d]	3.882[d]	6.047[d]	90.0	90.0	120.0			2.4[e]	
	Expt.	3.891[f]	3.891[f]	6.059[f]	90.0	90.0	120.0				
C-type Ce_2O_3	PBE+U	11.350	11.350	11.350	90.0	90.0	90.0	-6.06	1.25	2.65	4.03
	PBE0	11.200	11.200	11.200	90.0	90.0	90.0	-10.70	2.62	3.41	6.20
	HSE06	11.200	11.200	11.200	90.0	90.0	90.0	-10.62	2.64	2.68	5.49
	SM	11.165	11.165	11.165	90.0	90.0	90.0				
	Expt.	11.21[g]	11.21	11.21	90.0	90.0	90.0				
	Expt.	11.16[h]	11.16	11.16	90.0	90.0	90.0				
Ce_3O_5	PBE+U	6.910	6.910	9.697	90.0	90.0	60.0	-1.03	1.16	1.18	2.48
	PBE0	6.807	6.731	9.600	90.4	90.2	60.4	-0.29	2.51	1.94	4.74
	HSE06	6.809	6.732	9.602	90.4	90.2	60.4	7.71	2.53	1.18	3.99
	SM	6.803	6.822	9.591	90.0	90.0	60.1				
Ce_7O_{12}	PBE+U	6.902	6.893	6.826	98.8	100.1	99.5	-0.16	1.24	1.18	2.55
	PBE0	6.810	6.789	6.716	98.6	100.1	99.5	56.03	2.63	2.05	4.85
	HSE06	6.810	6.787	6.717	98.6	100.2	99.5	-0.07	2.64	1.28	4.09
	SM	6.841	6.684	6.889	98.7	100.8	99.3				
	Expt.	6.785[i]			99.42[i]						
$Ce_{11}O_{20}$	PBE+U	6.768	6.864	10.450	96.3	89.8	99.9	0.64	1.20	1.01	2.40
	PBE0	6.656	6.758	10.295	96.4	89.8	99.9	-0.07	2.64	1.77	4.58
	HSE06	6.657	6.758	10.295	96.4	89.8	99.9	0.21	2.66	1.01	3.83
	SM	6.753	6.759	10.298	96.0	90.5	99.6				
	Expt.	6.757[j]	6.732[j]	10.260[j]	96.22[j]	90.04[j]	99.80[j]				

Ce_6O_{11}[i]	PBE+U	6.815	11.796	13.063	90.0	100.1	90.0	0.02	1.26	1.00	2.44
	PBE0	6.701	11.655	12.847	89.9	79.9	90.3	26.07	2.98	1.67	4.81
	HSE06	6.653	11.529	12.754	90.0	100.0	90.0	-9.67	2.97	0.92	4.05
	SM	6.806	11.554	12.955	90.0	100.7	90.0				
	Expt.	6.781[k]	11.893[k]	15.823[k]		125.04[k]					
Ce_5O_9[l]	PBE+U	6.800	7.929	8.841	77.1	74.8	73.1	-29.16	1.19	1.12	2.51
	PBE0	6.633	7.744	8.639	77.1	75.0	73.2	-42.96	2.87	1.74	4.79
	HSE06	6.688	7.811	8.706	77.1	74.8	73.1	-40.32	2.89	0.98	4.05
	SM	6.841	7.782	8.703	77.0	74.0	73.4				

[a] Thermal expansion measurements extrapolated to 0 K.[1-4]

[b] Ref [5-6]

[c] Steady-state and ultrafast transient absorption spectra measurement on stoichiometric CeO_2 thin film. A revised optical band gap of 4 eV was proposed for bulk ceria, and the absorption tail below 4 eV was identified as the Urbach tail.[7]

[d] Ref. [8]

[e] Measured at 3 K.[9]

[f] Ref. [10]

[g] Ref. [11]

[h] Nonstoichiometric $CeO_{1.53-1.50}$.[12]

[i] Ref. [13]

[j] Initial structure adapted from Ref. [14]

[k] Measured at 855 °C.[15]

[l] Initial structure adapted from Materials Project (mp-760323).

Table S2. Shell-model potential parameters to model reduced CeO_{2-x}, shown in GULP-readable format. Ce4 and Ce3 indicates Ce^{4+} and Ce^{3+}, respectively.

```
Species
Ce4   core   -9.850000
Ce4   shel   13.850000
Ce3   core   -9.21790
Ce3   shel   12.21790
O     core    0.936345
O     shel   -2.936345
Buck
O     shel O     shel   22764.3000    0.149000   20.983768    0.00 25.00
O     shel Ce4   shel   1139.010286   0.417578   25.082349    0.00  4.80
O     shel Ce3   shel   2248.3906     0.341000   34.477930    0.00 25.00
Ce4   shel Ce4   shel   1.00000000    0.100000   30.481293    0.00 25.00
Ce3   shel Ce3   shel   1.00000000    0.100000   56.693757    0.00 25.00
Ce3   shel Ce4   shel   1.00000000    0.100000   39.621401    0.00 25.00
Lennard 12  6
O     shel  O     shel  10.0  0.0  0.000 25.000
O     shel  Ce4   shel  10.0  0.0  0.000  4.80
O     shel  Ce3   shel  10.0  0.0  0.000 25.000
Ce4   shel  Ce4   shel  10.0  0.0  0.000 25.000
Ce3   shel  Ce3   shel  10.0  0.0  0.000 25.000
Ce3   shel  Ce4   shel  10.0  0.0  0.000 25.000
Morse
O     shel  Ce4   shel  -1.15172262 .40000  4.53327  0.0000  0.000   4.80
Polynomial
1
O     shel  Ce4   shel  -1.077769  0.000000  0.000  0.000   4.80
Polynomial
5
O     shel  Ce4   shel -1952.35663530  1796.71132884  -658.98890407  &
120.42275309  -10.96459980  0.39794942  0.000  4.8  5.5
Spring
Ce4    1071.1845    100000.0
Ce3    799.14006    119228.97
O      53.022513    50000.0
```

Table S3. Computational details of hybrid QM/MM calculations of bulk IPs of CeO_{2-x} ordered phases.

(a) QM region		
ECP for Ce	Ref. [16] (28 core e^-)	
Basis set for Ce	Ref. [17]	
Basis set for O^{2-}	Def2-TZVP[18]	
DFT functional	Hybrid Meta-GGA functional BB1K[19]	
Code for QM calculations	NWChem[20-21]	
(b) QM/MM interface		
Formula of the embedding cation pseudopotentials	$U_p(r) = \frac{1}{r^2}(A_1\, r\, e^{-Z_1 r^2} + A_2\, r^2\, e^{-Z_2 r^2} + A_3\, r^2\, e^{-Z_3 r^2})$	
Type of Ion	Ce^{4+}	Ce^{3+}
A_1 (a.u.)	-49.817	-52.017
Z_1 (a.u.)	24.9589	30.9589
A_2 (a.u.)	60.247	61.917
Z_2 (a.u.)	2.85901	2.38801
A_3 (a.u.)	0.295877	0.381927
Z_3 (a.u.)	0.25855	0.32825
(c) MM regions		
Force field parameters	Table S2	
Code for MM calculations	GULP[22-23]	

Table S4. The calculated lattice constants a_0 and c_0, static dielectric constant ε_0, high-frequency dielectric constant ε_∞, elastic constants C_{11}, C_{33}, C_{12}, C_{13}, and C_{44}, bulk modulus B_0, lattice energy ΔH_L, Born effective charge Z^*, and vibrational frequencies at the Γ point of *A*-type Ce_2O_3 based on the interatomic-potential calculations using parameters presented in Table S2, compared with experimental and DFT results.

	Interatomic potential*	Expt.	HSE06*	HSE06[b]	PBE+*U* (U = 5.0 eV)[c]
a_0 (Å)	3.8509	3.88166[a]	3.87	3.87	3.93
c_0 (Å)	6.0845	6.04731[a]	6.06	6.06	6.15
$\varepsilon_{0\ a/c}$	19.03/21.32	22.9[a]			
$\varepsilon_{\infty\ a/c}$	4.40/4.10		4.31/4.17		
C_{11}	278.03			237.5	211
C_{33}	196.45			151.4	142
C_{12}	138.12			137.4	123
C_{13}	108.71			94.4	83
C_{44}	93.23			72.1	62
B_0 (GPa)	155.31	111[d]		135.8	122
ΔH_L (eV)	-129.41	-131.2[e]			
$Z^*_{Ce\_a/c}$	3.83/3.73		4.12/3.75		
$Z^*_{O1\_a/c}$	-2.31/-2.34		-2.69/-2.55		
$Z^*_{O2\_a/c}$	-3.05/-2.78		-2.78/-2.48		
E_g (cm^{-1})	123.22	103[f]		107	
A_{1g} (cm^{-1})	221.55	189[f]		193	
E_g (cm^{-1})	417.30	409[f]		423	
A_{1g} (cm^{-1})	438.25	409[f]		424	
E_u (cm^{-1})	221.64			205	
A_{2u} (cm^{-1})	223.22			218	
E_u (cm^{-1})	410.99			387	
A_{2u} (cm^{-1})	483.10			467	

* Present work

[a] Measured at 3 K in Ref. [8]

[b] Ref. [24]

[c] Ref. [25]

[d] Ref. [26]

[e] Ref. [27]

[f] Ref. [28]

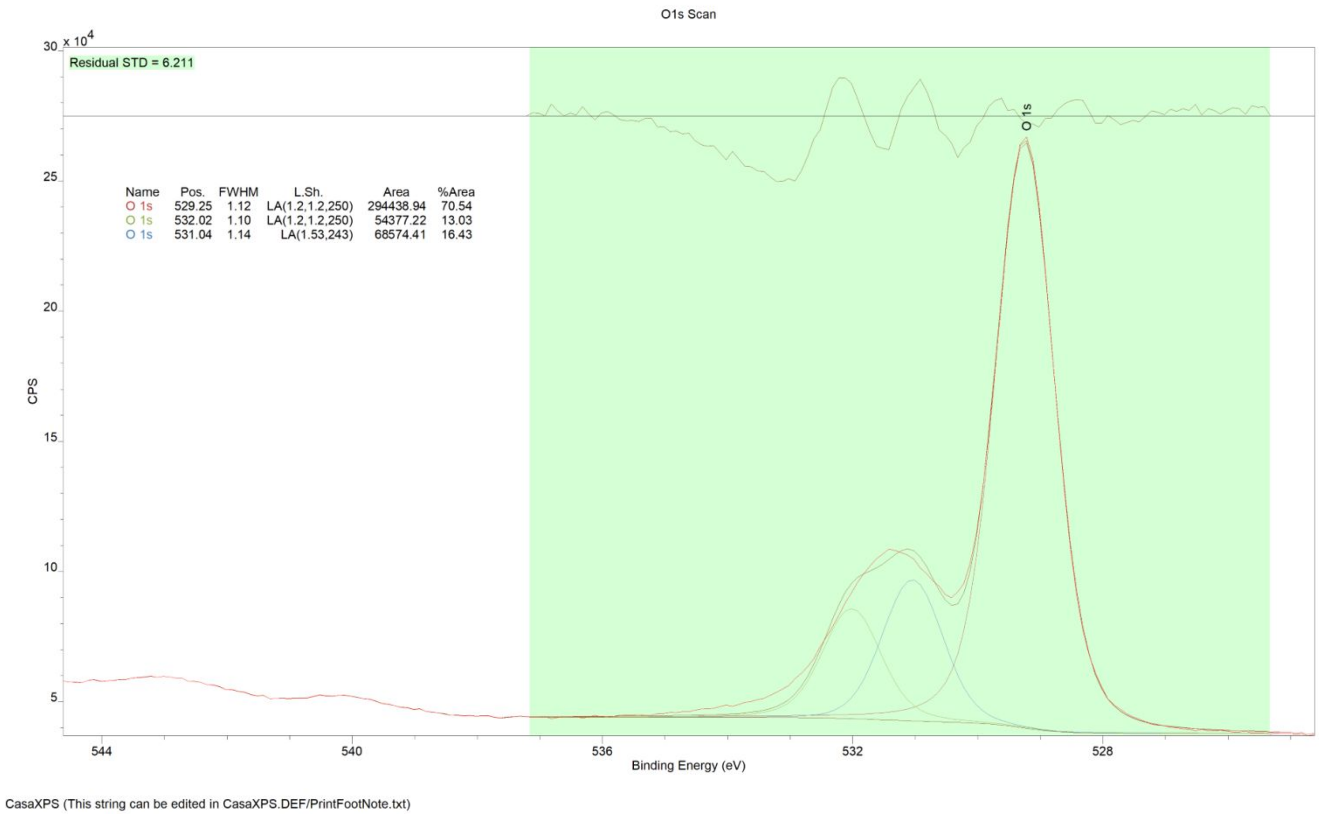

Figure S1. Deconvolution of the O 1*s* XPS spectrum.

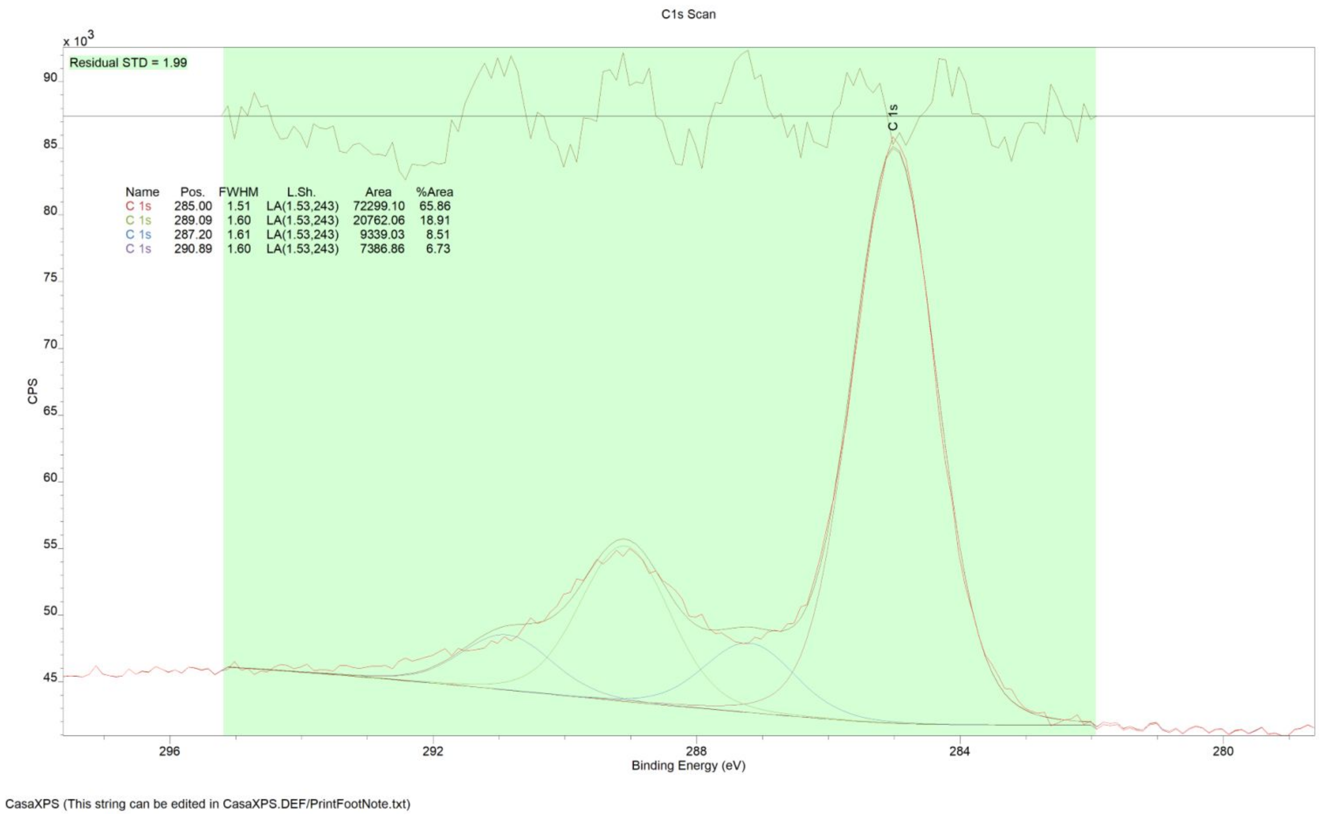

Figure S2. Deconvolution of the C 1*s* XPS spectrum.

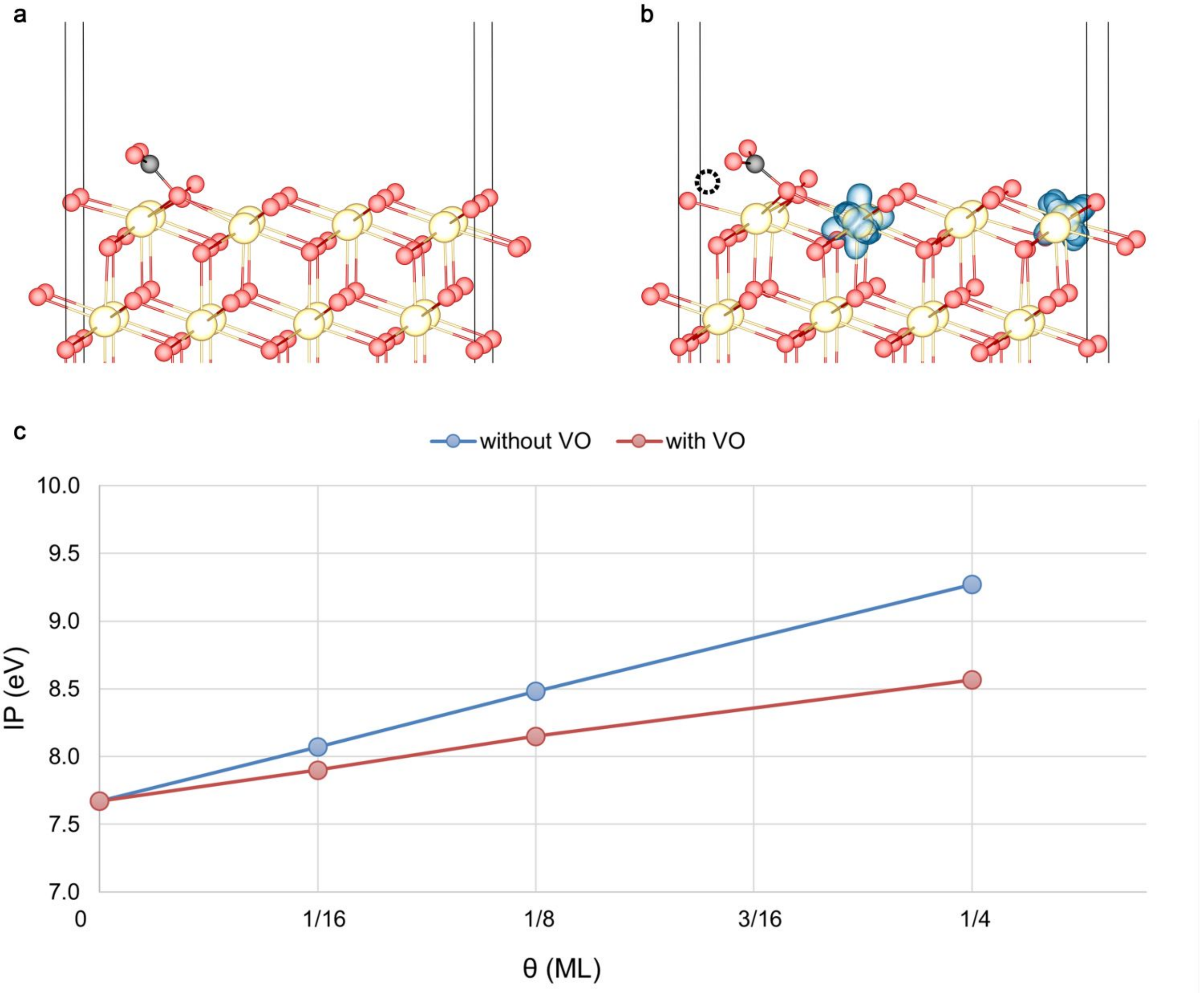

Figure S3. Snapshots of carbonate species formed on the (a) pristine and (b) defective CeO_2(111) surfaces. (c) Comparison of the calculated ionisation potentials of pristine and defective CeO_2(111) with different coverages of surface carbonate species.

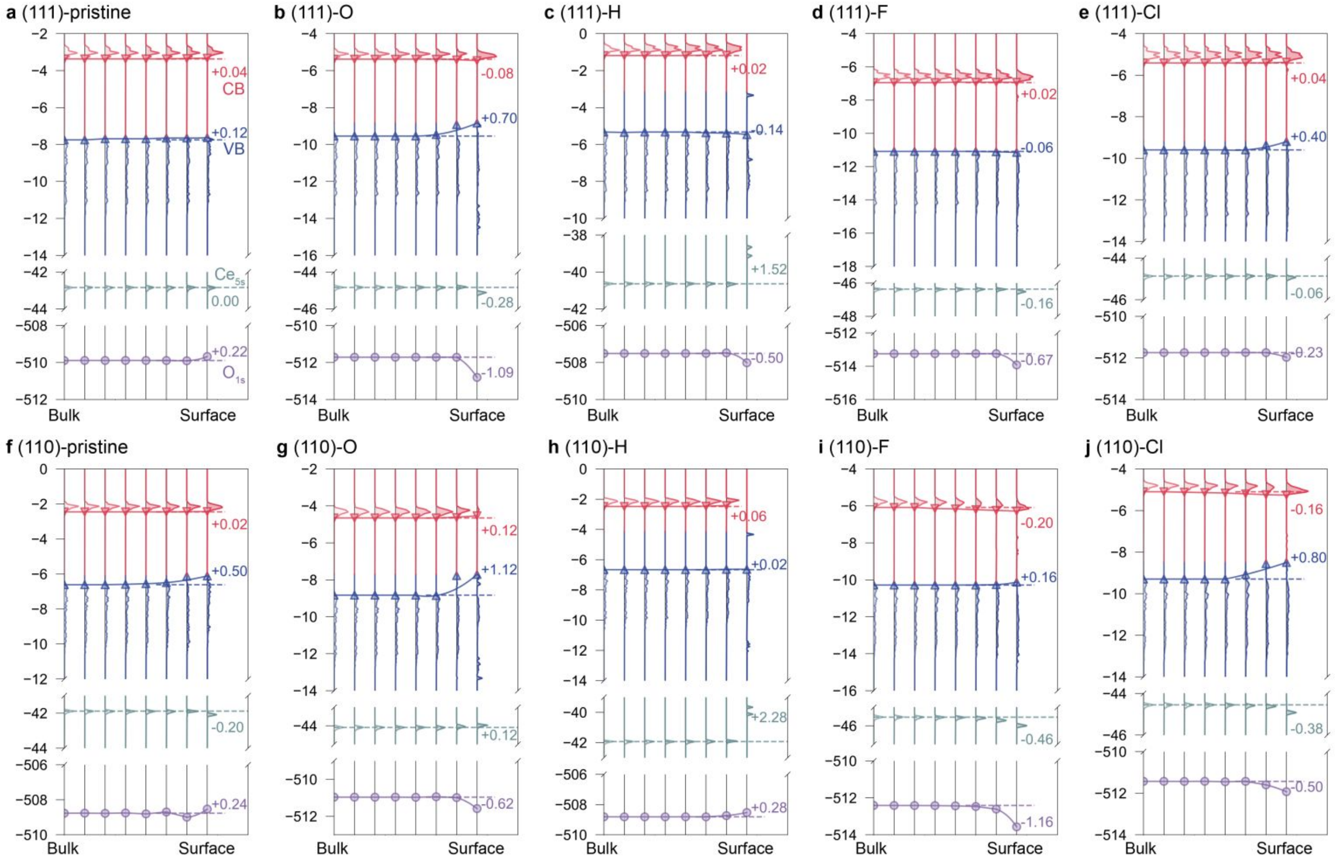

Figure S4. Layer-by-layer density of states (DOS) of (a-e) CeO_2(111) and (f-j) CeO_2(110) with different types of surface adsorbates at a coverage of 1 monolayer with respect to the vacuum level.

References

1. Stecura, S.; Campbell, W. J., *Thermal expansion and phase inversion of rare-earth oxides*. US Department of the Interior, Bureau of Mines: 1961; Vol. 5847.
2. GUPTA, M. L.; Singh, S., Thermal Expansion of CeO_2, Ho_2O_3, and Lu_2O_3 from 100° to 300° K by an X-Ray Method. *J. Am. Ceram. Soc.* **1970,** *53* (12), 663-665.
3. Rossignol, S.; Gérard, F.; Mesnard, D.; Kappenstein, C.; Duprez, D., Structural changes of Ce–Pr–O oxides in hydrogen: a study by in situ X-ray diffraction and Raman spectroscopy. *J. Mater. Chem.* **2003,** *13* (12), 3017-3020.
4. Yashima, M.; Kobayashi, S.; Yasui, T., Crystal structure and the structural disorder of ceria from 40 to 1497 °C. *Solid State Ionics* **2006,** *177* (3-4), 211-215.
5. Wuilloud, E.; Delley, B.; Schneider, W.-D.; Baer, Y., Spectroscopic Evidence for Localized and Extended f-Symmetry States in CeO_2. *Phys. Rev. Lett.* **1984,** *53* (2), 202.
6. Graciani, J.; Márquez, A. M.; Plata, J. J.; Ortega, Y.; Hernández, N. C.; Meyer, A.; Zicovich-Wilson, C. M.; Sanz, J. F., Comparative study on the performance of hybrid DFT functionals in highly correlated oxides: the case of CeO_2 and Ce_2O_3. *J. Chem. Theory Comput.* **2011,** *7* (1), 56-65.
7. Pelli Cresi, J. S.; Di Mario, L.; Catone, D.; Martelli, F.; Paladini, A.; Turchini, S.; D'Addato, S.; Luches, P.; O'Keeffe, P., Ultrafast Formation of Small Polarons and the Optical Gap in CeO_2. *J. Phys. Chem. Lett.* **2020,** *11* (14), 5686-5691.
8. Kolodiazhnyi, T.; Sakurai, H.; Avdeev, M.; Charoonsuk, T.; Lamonova, K.; Pashkevich, Y. G.; Kennedy, B., Giant magnetocapacitance in cerium sesquioxide. *Phys. Rev. B* **2018,** *98* (5), 054423.
9. Prokofiev, A.; Shelykh, A.; Melekh, B., Periodicity in the band gap variation of Ln_2X_3 (X= O, S, Se) in the lanthanide series. *J. Alloys Compd.* **1996,** *242* (1-2), 41-44.
10. Bärnighausen, H.; Schiller, G., The crystal structure of A-Ce_2O_3. *J. Less-Common Met.* **1985,** *110* (1-2), 385-390.
11. Perrichon, V.; Laachir, A.; Bergeret, G.; Fréty, R.; Tournayan, L.; Touret, O., Reduction of cerias with different textures by hydrogen and their reoxidation by oxygen. *J. Chem. Soc., Faraday Trans.* **1994,** *90* (5), 773-781.
12. Adachi, G.-y.; Imanaka, N., The Binary Rare Earth Oxides. *Chem. Rev.* **1998,** *98* (4), 1479-1514.
13. Kümmerle, E.; Heger, G., The structures of C–$Ce_2O_{3+\delta}$, Ce_7O_{12}, and $Ce_{11}O_{20}$. *J. Solid State Chem.* **1999,** *147* (2), 485-500.
14. Shoko, E.; Smith, M.; McKenzie, R. H., Charge distribution near bulk oxygen vacancies in cerium oxides. *J. Phys.: Condens. Matter* **2010,** *22* (22), 223201.
15. Sørensen, O. T., Thermodynamic studies of the phase relationships of nonstoichiometric cerium oxides at higher temperatures. *J. Solid State Chem.* **1976,** *18* (3), 217-233.
16. Dolg, M.; Stoll, H.; Preuss, H., Energy-adjusted ab initio pseudopotentials for the rare earth elements. *J. Chem. Phys.* **1989,** *90* (3), 1730-1734.
17. Desmarais, J. K.; Erba, A.; Dovesi, R., Generalization of the periodic LCAO approach in the CRYSTAL code to g-type orbitals. *Theor. Chem. Acc.* **2018,** *137* (2), 1-11.

18. Weigend, F.; Ahlrichs, R., Balanced basis sets of split valence, triple zeta valence and quadruple zeta valence quality for H to Rn: Design and assessment of accuracy. *Phys. Chem. Chem. Phys.* **2005,** *7* (18), 3297-3305.
19. Zhao, Y.; Lynch, B. J.; Truhlar, D. G., Development and assessment of a new hybrid density functional model for thermochemical kinetics. *J. Phys. Chem. A* **2004,** *108* (14), 2715-2719.
20. Valiev, M.; Bylaska, E. J.; Govind, N.; Kowalski, K.; Straatsma, T. P.; Van Dam, H. J.; Wang, D.; Nieplocha, J.; Apra, E.; Windus, T. L., NWChem: A comprehensive and scalable open-source solution for large scale molecular simulations. *Comput. Phys. Commun.* **2010,** *181* (9), 1477-1489.
21. Apra, E.; Bylaska, E. J.; De Jong, W. A.; Govind, N.; Kowalski, K.; Straatsma, T. P.; Valiev, M.; van Dam, H. J.; Alexeev, Y.; Anchell, J., NWChem: Past, present, and future. *J. Chem. Phys.* **2020,** *152* (18), 184102.
22. Gale, J. D., GULP: A computer program for the symmetry-adapted simulation of solids. *J. Chem. Soc., Faraday Trans.* **1997,** *93* (4), 629-637.
23. Gale, J. D.; Rohl, A. L., The general utility lattice program (GULP). *Mol. Simul.* **2003,** *29* (5), 291-341.
24. Brugnoli, L.; Ferrari, A. M.; Civalleri, B.; Pedone, A.; Menziani, M. C., Assessment of density functional approximations for highly correlated oxides: the case of CeO_2 and Ce_2O_3. *J. Chem. Theory Comput.* **2018,** *14* (9), 4914-4927.
25. Weck, P. F.; Kim, E., Assessing Hubbard-corrected AM05+ U and PBEsol+ U density functionals for strongly correlated oxides CeO_2 and Ce_2O_3. *Phys. Chem. Chem. Phys.* **2016,** *18* (38), 26816-26826.
26. Lipp, M.; Jeffries, J.; Cynn, H.; Klepeis, J.-H. P.; Evans, W.; Mortensen, D.; Seidler, G.; Xiao, Y.; Chow, P., Comparison of the high-pressure behavior of the cerium oxides Ce_2O_3 and CeO_2. *Phys. Rev. B* **2016,** *93* (6), 064106.
27. Haynes, W. M., *CRC handbook of chemistry and physics*. CRC press: 2014.
28. Schilling, C.; Hofmann, A.; Hess, C.; Ganduglia-Pirovano, M. V. n., Raman spectra of polycrystalline CeO_2: a density functional theory study. *J. Phys. Chem. C* **2017,** *121* (38), 20834-20849.
